# Determining the role of novel metabolic pathways in driving intracranial pressure reduction after weight loss

**DOI:** 10.1093/braincomms/fcad272

**Published:** 2023-10-18

**Authors:** Zerin Alimajstorovic, James L Mitchell, Andreas Yiangou, Thomas Hancox, Andrew D Southam, Olivia Grech, Ryan Ottridge, Catherine L Winder, Abd A Tahrani, Tricia M Tan, Susan P Mollan, Warwick B Dunn, Alexandra J Sinclair

**Affiliations:** Institute of Metabolism and Systems Research, College of Medical and Dental Sciences, University of Birmingham, Birmingham B15 2TT, UK; Institute of Metabolism and Systems Research, College of Medical and Dental Sciences, University of Birmingham, Birmingham B15 2TT, UK; Department of Neurology, University Hospitals Birmingham NHS Foundation Trust, Queen Elizabeth Hospital, Birmingham B15 2GW, UK; Institute of Metabolism and Systems Research, College of Medical and Dental Sciences, University of Birmingham, Birmingham B15 2TT, UK; Department of Neurology, University Hospitals Birmingham NHS Foundation Trust, Queen Elizabeth Hospital, Birmingham B15 2GW, UK; School of Biosciences, College of Life and Environmental Sciences, University of Birmingham, Birmingham B15 2TT, UK; School of Biosciences, College of Life and Environmental Sciences, University of Birmingham, Birmingham B15 2TT, UK; Institute of Metabolism and Systems Research, College of Medical and Dental Sciences, University of Birmingham, Birmingham B15 2TT, UK; Birmingham Clinical Trials Unit, College of Medical and Dental Sciences, University of Birmingham, Birmingham B15 2TT, UK; School of Biosciences, College of Life and Environmental Sciences, University of Birmingham, Birmingham B15 2TT, UK; Department of Biochemistry and Systems Biology, Institute of Systems, Molecular, and Integrative Biology, University of Liverpool, Liverpool L3 5TR, UK; Institute of Metabolism and Systems Research, College of Medical and Dental Sciences, University of Birmingham, Birmingham B15 2TT, UK; Centre for Endocrinology, Diabetes and Metabolism, Birmingham Health Partners, Birmingham B15 2TT, UK; Section of Endocrinology and Investigative Medicine, Department of Metabolism, Digestion and Reproduction, Faculty of Medicine, Imperial College London, London SW7 2BX, UK; Institute of Metabolism and Systems Research, College of Medical and Dental Sciences, University of Birmingham, Birmingham B15 2TT, UK; Birmingham Neuro-Ophthalmology, University Hospitals Birmingham, Queen Elizabeth Hospital, Birmingham B15 2GW, UK; Institute of Metabolism and Systems Research, College of Medical and Dental Sciences, University of Birmingham, Birmingham B15 2TT, UK; School of Biosciences, College of Life and Environmental Sciences, University of Birmingham, Birmingham B15 2TT, UK; Department of Biochemistry and Systems Biology, Institute of Systems, Molecular, and Integrative Biology, University of Liverpool, Liverpool L3 5TR, UK; Institute of Metabolism and Systems Research, College of Medical and Dental Sciences, University of Birmingham, Birmingham B15 2TT, UK; Centre for Endocrinology, Diabetes and Metabolism, Birmingham Health Partners, Birmingham B15 2TT, UK; Birmingham Neuro-Ophthalmology, University Hospitals Birmingham, Queen Elizabeth Hospital, Birmingham B15 2GW, UK

**Keywords:** pseudotumor cerebri, metabolomics, meal stimulation, bariatric surgery

## Abstract

Idiopathic intracranial hypertension, a disease classically occurring in women with obesity, is characterized by raised intracranial pressure. Weight loss leads to the reduction in intracranial pressure. Additionally, pharmacological glucagon-like peptide-1 agonism reduces cerebrospinal fluid secretion and intracranial pressure. The potential mechanisms by which weight loss reduces intracranial pressure are unknown and were the focus of this study. Meal stimulation tests (fasted plasma sample, then samples at 15, 30, 60, 90 and 120 min following a standardized meal) were conducted pre- and post-bariatric surgery [early (2 weeks) and late (12 months)] in patients with active idiopathic intracranial hypertension. Dynamic changes in gut neuropeptides (glucagon-like peptide-1, gastric inhibitory polypeptide and ghrelin) and metabolites (untargeted ultra-high performance liquid chromatography-mass spectrometry) were evaluated. We determined the relationship between gut neuropeptides, metabolites and intracranial pressure. Eighteen idiopathic intracranial hypertension patients were included [Roux-en-Y gastric bypass (RYGB) *n* = 7, gastric banding *n* = 6 or sleeve gastrectomy *n* = 5]. At 2 weeks post-bariatric surgery, despite similar weight loss, RYGB had a 2-fold (50%) greater reduction in intracranial pressure compared to sleeve. Increased meal-stimulated glucagon-like peptide-1 secretion was observed after RYGB (+600%) compared to sleeve (+319%). There was no change in gastric inhibitory polypeptide and ghrelin. Dynamic changes in meal-stimulated metabolites after bariatric surgery consistently identified changes in lipid metabolites, predominantly ceramides, glycerophospholipids and lysoglycerophospholipids, which correlated with intracranial pressure. A greater number of differential lipid metabolites were observed in the RYGB cohort at 2 weeks, and these also correlated with intracranial pressure. In idiopathic intracranial hypertension, we identified novel changes in lipid metabolites and meal-stimulated glucagon-like peptide-1 levels following bariatric surgery which were associated with changes in intracranial pressure. RYGB was most effective at reducing intracranial pressure despite analogous weight loss to gastric sleeve at 2 weeks post-surgery and was associated with more pronounced changes in these metabolite pathways. We suggest that these novel perturbations in lipid metabolism and glucagon-like peptide-1 secretion are mechanistically important in driving a reduction in intracranial pressure following weight loss in patients with idiopathic intracranial hypertension. Therapeutic targeting of these pathways, for example with glucagon-like peptide-1 agonist infusion, could represent a therapeutic strategy.

## Teaser

In idiopathic intracranial hypertension, Alimajstorovic *et al*. identified novel changes in lipid metabolites (ceramides, glycerophospholipids and lysoglycerophospholipids) and meal-stimulated glucagon-like peptide-1 levels following bariatric surgery which were associated with changes in intracranial pressure, most notably following Roux-en-Y gastric bypass.

## Graphical Abstract

**Graphical abstract:**
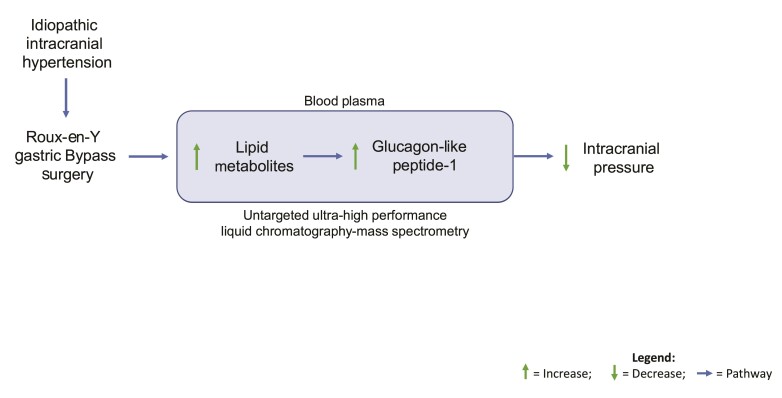

## Introduction

Idiopathic intracranial hypertension (IIH) is a disease of raised intracranial pressure (ICP).^1,2^ Symptoms of IIH include disabling daily headaches and visual disturbances, with papilloedema leading to permanent visual loss in up to 40% of patients.^1,3-5^ The underlying pathogenesis of IIH is not fully understood but the disease occurs predominantly in women with obesity^6,7^ and the incidence of IIH is increasing in line with country-specific obesity rates.^8,9^ IIH disease activity, as measured by ICP, correlates closely with truncal adiposity.^10,11^ Weight loss is therapeutic in IIH and reduces ICP.^12,13^ However, the mechanism by which weight loss reduces ICP is not known. In IIH, there is systemic metabolic dysregulation in excess of that predicted by obesity, including insulin resistance and hyperleptinaemia.^10,14^ In addition, a distinct profile of androgen excess and glucocorticoid dysregulation have been noted.^15-17^ These factors may drive the increased risk of cardiovascular disease (CVD), type 2 diabetes mellitus (T2D),^9^ obstructive sleep apnoea,^14^ reduced fertility, gestational diabetes and pre-eclampsia^18,19^ compared with age, sex, body mass index (BMI) matched populations, in IIH. Additionally, metabolic dysregulation has been noted in association with the severe headaches observed in IIH.^20-22^

The gut neuropeptide glucagon-like peptide-1 (GLP-1) is of interest in IIH. GLP-1 is an incretin hormone secreted in the gut and is known to stimulate insulin secretion and inhibit glucagon release.^23,24^ GLP-1 is also synthesized in neurons of the nucleus tractus solitarius that project to the hypothalamus^25^ and promotes satiety and weight loss.^26^*In vivo* data has identified GLP-1 receptor (GLP-1R) expression in the human and rodent choroid plexus.^27,28^ The GLP-1 receptor agonist, Exenatide, directly reduces cerebrospinal fluid (CSF) secretion and ICP *in vivo*.^27^ Consequently, GLP receptor agonism has been investigated as a potential target for treating conditions with raised ICP such as IIH. A randomized double-blind placebo-controlled trial in patients with active IIH demonstrated that Exenatide significantly reduced ICP in IIH at 2.5 h, 24 h and at 12 weeks,^29^ suggesting a weight-independent and direct effect of GLP-1R agonsim on ICP in IIH.

Bariatric surgery has been shown to significantly reduce ICP in association with the amount of weight loss, in IIH, in a randomized clinical trial (IIH:WT).^12,30^ Weight loss and ICP reduction were most pronounced in those undergoing Roux-en-Y gastric bypass (RYGB).^7^ Different types of bariatric surgery are known to have differing effects on GLP-1 secretion with the most pronounced changes occurring in those undergoing RYGB surgery, a procedure which bypasses food to the mid/distal jejunum and exposes L-cells to nutrients which triggers a sharp rise in GLP-1, oxyntomodulin and peptide YY.^31,32^ In contrast, sleeve gastrectomy leads to accelerated gastric emptying which also triggers increased GLP-1 secretion, but less marked peptide YY secretion.^31-34^

In the IIH bariatric surgery trial (IIH:WT), it was observed that the amount of weight loss was significantly correlated with the degree of reduction in ICP.^30^ Of interest, however, was the observation that ICP was very rapidly reduced (at 2 weeks) after bariatric surgery, and this appeared to be predominantly independent of weight loss as only relatively small changes in body weight had occurred at this time point.^12^ This observation is akin to the early improvements in glycaemic control noted at 2 weeks post-bariatric surgery in patients with T2D, which were predominantly noted among those undergoing RYGB surgery. In T2D, this phenomenon has been linked to the increased post-prandial GLP-1, oxyntomodulin and peptide YY secretion.^31,32^

In this study, we hypothesized that the therapeutic efficacy of weight loss in IIH may be driven by changes in metabolism and gut neuropeptides such as GLP-1. We sought to gain an understanding of the mechanisms underlying the reduction in ICP in people with IIH following bariatric surgery through evaluation of gut neuropeptide and metabolic profiles, by applying untargeted ultra-high performance liquid chromatography-mass spectrometry (UHPLC-MS) metabolomic analysis, over the course of a meal stimulation test. We then determined if the type of bariatric surgery had a differential effect on metabolism [at 2 weeks (early) and 12 months (late)] following surgery. Finally, we aimed to study the relationship between gut neuropeptides, metabolites and ICP.

## Materials and methods

### Study type

This was a pre-planned sub-analysis of the IIH:WT randomized control trial.^35^ This trial identified and recruited IIH subjects from neurology and ophthalmology clinics from seven UK National Health Service hospitals. These sites as well as the clinical trial protocol and results have been published elsewhere^12,35,36^ and received ethical approval from the National Research Ethics Committee West Midlands—The Black Country REC (14/WM/0011, Dudley, UK). All participants provided written informed consent.

### Trial details

IIH:WT was funded by the National Institute for Health and Care Research (NIHR-CS-011-028) and registered with ClinicalTrials.gov: NCT02124486.

### Study population

The eligibility criteria for the main IIH:WT have previously been published.^35^ In brief, this included women aged 18–55 years, with a BMI ≥ 35 kg/m^2^ and active IIH (lumbar puncture opening pressure >25 cmCSF and Frisén papilloedema grade ≥1).^12,35^ For this sub-study, additional eligibility criteria included being randomized to the bariatric surgery arm of the trial (RYGB, gastric sleeve or gastric band^12,35^) and consenting to undergo meal stimulation testing.

### Clinical assessments

The IIH participants attended trial visits at baseline, 2 weeks post-surgery and at 12 months, according to the published protocols.^35^ All participants underwent detailed medical history and clinical examination including a pregnancy test. BMI was calculated from weight and height using the following formula: BMI = [weight (kg)/height (m)^2^]. Visual tests performed included the perimetric mean deviation (PMD) using Humphrey 24-2 Swedish Interactive Thresholding Algorithm (SITA)^37^ central threshold automated perimetry and spectral domain optical coherence tomography (OCT; Spectralis, Heidelberg Engineering) imaging to evaluate the average peripapillary retinal nerve fibre layer (RNFL), a measure of papilloedema.^38^ Monthly headache days and headache severity were recorded using headache diaries and headache-associated disability was measured using the headache impact test-6 score (HIT-6). Lumbar punctures were conducted at baseline, 2 weeks post-surgery and at 12 months using a standardized procedure in the lateral decubitus position under ultrasound guidance with lumbar puncture opening pressure recorded.^12,35^

### Meal stimulation

Meal stimulation tests were performed at baseline; 2 weeks post-surgery and at 12 months in all IIH patients as previously described.^39^ Meal stimulation testing was conducted following an overnight fast from midnight. In brief, baseline samples were collected from all patients before a standardized meal was administered (Fortisip 200 ml, Cat No. 18499 309-2129, Nutricia, UK) (Composition of product found in Supplementary Table 1). A timed series of blood samples were collected at 15, 30, 60, 90 and 120 min following the standardized meal. Blood for gut hormones was collected in ethylenediamin tetra-acetic acid tubes (catalogue number: 456011, Greiner Bio-One Ltd, UK) containing 40 µL dipeptidyl peptidase 4 inhibitor (catalogue number: DPP4-010, Merck-Millipore UK Ltd, UK). All blood samples collected were then centrifuged (10 min at 1500 *g* at 4°C), aliquoted as plasma in microcentrifuge tubes containing 2.5 µL protease inhibitor cocktail (catalogue number: P8340, Sigma, UK) and stored at −80°C. All samples processed only underwent a single freeze–thaw cycle.

### Gut neuropeptide analysis

Active GLP-1, ghrelin and gastric inhibitory polypeptide (GIP) plasma samples were assayed together using a customized multiplex magnetic bead-based immunoassay (MILLIPLEX® MAP Human Metabolic Hormone Magnetic Bead Panel (Catalogue # HMHEMAG-34K), Merck-Millipore, Germany) according to the manufacturer’s instructions and read using a Luminex MagPix® analyser. Minimum detectable concentrations were 0.4 pmol/L for active GLP-1, 3.9 pmol/L for ghrelin and 0.1 pmol/L for GIP. The precision of intra-assay and inter-assay (%CV) are <10 and <15, respectively, for all three assayed gut hormones.^40^

### Metabolomics analysis

The detailed metabolomic analysis methods are described in the Supplementary material. In summary, metabolites present in plasma samples were extracted using a monophasic organic solvent (50/50 acetonitrile/water or isopropanol) and analysed by applying UHPLC-MS assays (HILIC for water-soluble metabolites and lipidomics for lipid metabolites) in positive and negative ion modes. Raw data processing was performed using XCMS. MS1, MS/MS and retention time data were applied to structurally annotate metabolites. Univariate analysis (one-way repeated measures ANOVA), Spearman’s rank correlation analysis, hierarchical cluster analysis and pathway enrichment analysis were performed in MetaboAnalyst v5.0.

### Statistical analysis

The gut neuropeptide data was analysed by calculating the total area under the curve (AUC) from fasted samples at baseline, 2 weeks post-surgery and 12-month time points as well as from the time of ingestion of the mixed meal to the post-prandial phase (0–120 min) at all time points. Statistical analysis was performed using GraphPad Prism 9.4.1 9 (GraphPad software).

For the metabolomics data analysis, statistical and pathway enrichment analysis was performed in MetaboAnalyst v5.0.^41^ For statistical analysis, data were normalized to total sample response and log10 transformed. Statistical analysis applied a one-way repeated measures ANOVA, *q* < 0.05 for the baseline meal-stimulated metabolite changes, over the meal stimulation test, and a two-way repeated measures ANOVA (*P* < 0.005) for metabolite time (2 weeks and 12 months) and phenotype interactions (type of surgery). Pathway enrichment analysis applied pathway analysis, hypergeometric test (enrichment method), relative-betweenness centrality (topology analysis) and *Mus musculus* (KEGG) as the pathway library.

For the correlation analysis of ICP with metabolites, we performed Spearman’s rank correlation analysis. The abundance of each metabolite detected (time point 0, the start of the meal stimulation test) was correlated with ICP measured at 2 weeks post-surgery. Separately, the changes in ICP (2 weeks post-surgery minus baseline values) were also compared to changes in abundances of each metabolite detected (fasted metabolites at time point 0 of the meal stimulation test). These comparisons were performed for RYGB only patients and for the combined set of sleeve and RYGB patients. Correlation of sleeve data was not performed because data was only available for three sleeve patients.

Hierarchical Clustering Heatmaps were constructed in MetaboAnalyst using the following parameters: Features (autoscaled), distance measure (Euclidean), clustering method (Ward) and samples (not clustered). The minimum or maximum peak response in comparison to the baseline sample (whichever was the higher) reported across the 120-min period of collection post-meal was used.

## Results

### Patient characteristics and phenotypic changes in weight, BMI and ICP

**Figure 1:**
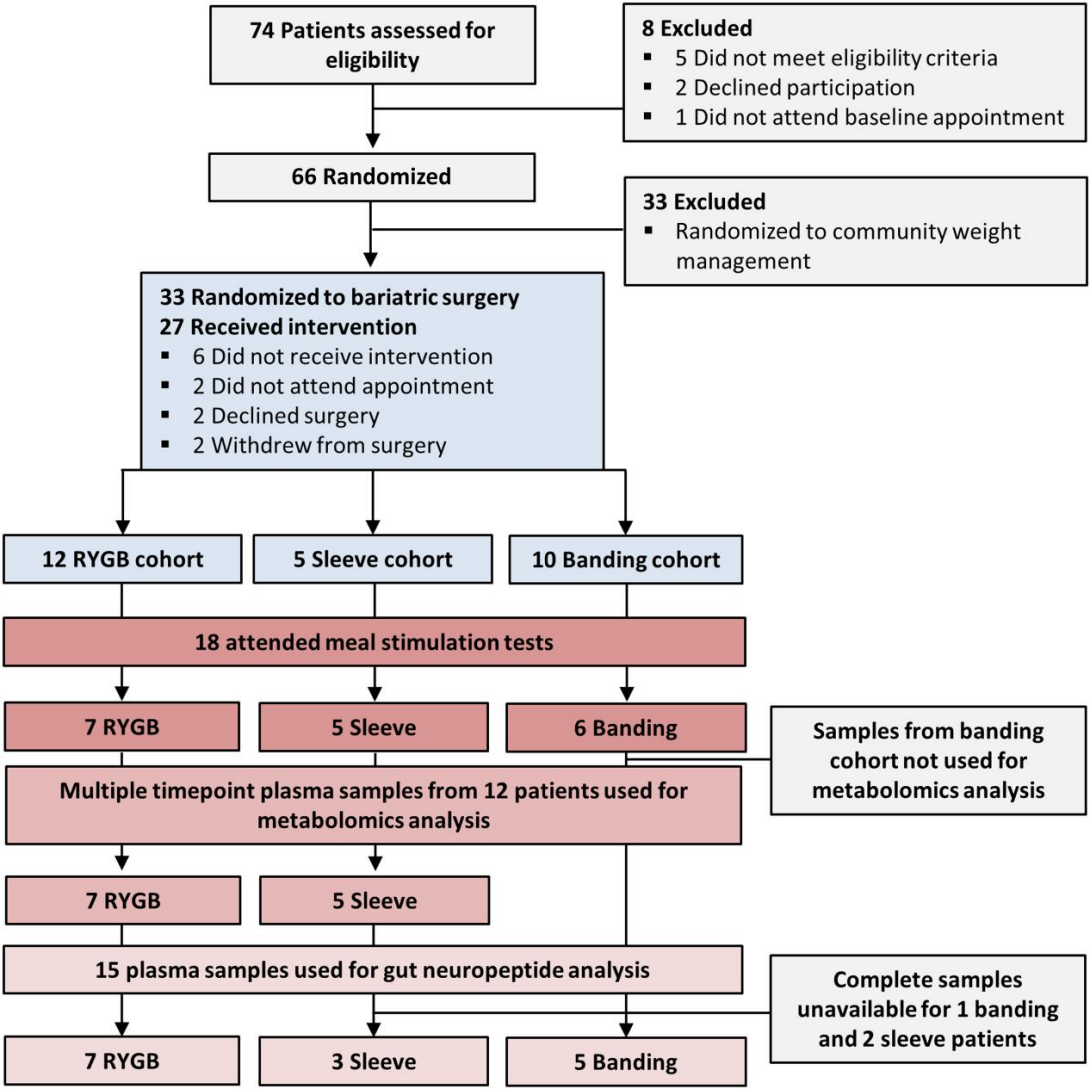
**Consort diagram**. Thirty-three participants out of 66 recruited were randomized to a bariatric surgery, of which 27 received an intervention. All 18 participants were female and consented to receive a meal stimulation test (RYGB *n* = 7, banding *n* = 6, sleeve *n* = 5).

For this study, 33 participants out of 66 recruited were randomized to a bariatric surgery, of which 27 received an intervention (Fig. 1). Eighteen participants consented to receive a meal stimulation test (RYGB *n* = 7, banding *n* = 6, sleeve *n* = 5). The baseline characteristics were typical for a population of people with active IIH (Table 1).

**Table 1:** Baseline characteristics of the study subject combined and split by surgery type (RYGB, sleeve and band)

Baseline characteristics	All surgeries (*n* = 18)	RYGB (*n* = 7)	Sleeve (*n* = 5)	Band (*n* = 6)
Age (years)	30.8 (6.9)	32.7 (9.2)	29.4 (6.9)	29.8 (4.0)
Weight (kg)	111.9 (20.4)	115.0 (27.3)	113.1 (19.8)	107.2 (13.0)
BMI (kg/m^2^)	42.9 (7.0)	45.5 (9.6)	42.2 (4.3)	40.4 (4.8)
ICP (cmCSF)	35.6 (4.5)	35.6 (5.6)	35.6 (4.7)	35.5 (3.6)
Systolic blood pressure (mmHg)	124.8 (18.4)	131.8 (17.3)	126.2 (10.4)	115.4 (23.1)
PMD (worst eye) (dB)	−4.3 (3.9)	−4.0 (3.9)	−4.8 (6.0)	−4.4 (1.1)
Papilloedema measured by OCT global RNFL (worst eye) (µm)	160.1 (128.3)	196.6 (191.4)	136.0 (61.8)	133.0 (55.3)
Monthly headache days	23.6 (6.6)	24.6 (5.9)	21.6 (8.3)	24.0 (6.7)
Frisén grading of papilloedema (worst eye)	2.2 (1.0)	2.3 (1.0)	2.5 (1.0)	1.8 (1.3)

Data presented as mean ± SD.

*n*, number.

### Relationship between degree of weight loss and intracranial pressure reduction

Initially, we evaluated the degree of ICP reduction from each bariatric surgery type in relation to the amount of weight loss at 2 weeks (Table 2). The analysis at 12 months has been previously published in the entire IIH:WT study and the results pertaining to the individuals included in this study are shown in Table 2.^7^ We then evaluated the data at 2 weeks post-surgery. At 2 weeks, the cohort undergoing RYGB demonstrated the greatest reduction in ICP compared to other surgical types (RYGB −12.7 ± 10.5 cmCSF; gastric sleeve −6.4 ± 8.8 cmCSF; gastric banding −7.3 ± 1.1 cmCSF), with a 50% greater reduction in RYGB compared to sleeve. This occurred despite similar weight loss in the RYGB and sleeve cohorts at 2 weeks (RYGB −10.4 ± 7.4 kg; gastric sleeve −9.9 ± 5.2 kg). The gastric banding group lost the least amount of weight at 2 weeks and had the least reduction in ICP (Table 2) (all surgeries: ΔWeight (kg) versus ΔICP (mmH_2_O); *r* = 0.287, *P* = 0.263) (Supplementary Fig. 1A). At 12 months, a similar but less pronounced pattern was observed with those undergoing RYGB having a 16.0% greater reduction in ICP compared to sleeve (all surgeries: ΔWeight (kg) versus ΔICP (mmH_2_O); *r* = 0.587, *P* = 0.01) (Supplementary Fig. 1B).

**Table 2:** Absolute and delta change (Δ) values for age, BMI, ICP and weight

	Baseline mean (SD)				2 weeks post-surgery mean (SD)				12 months mean (SD)			
	All surgeries (*n* = 18)	RYGB (*n* = 7)	Sleeve (*n* = 5)	Band (*n* = 6)	All surgeries (*n* = 18)	RYGB (*n* = 7)	Sleeve (*n* = 5)	Band (*n* = 6)	All surgeries (*n* = 18)	RYGB (*n* = 7)	Sleeve (*n* = 5)	Band (*n* = 6)
Age (years)	30.8 (6.9)	32.7 (9.2)	29.4 (6.9)	29.8 (4.0)								
BMI (kg/m^2^)	42.9 (7.0)	45.5 (9.6)	42.2 (4.3)	40.4 (4.8)	38.7 (6.3)	40.2 (8.6)	37.9 (5.3)	37.6 (4.2)	32.8 (6.8)	32.3 (9.8)	31.0 (3.6)	34.9 (4.8)
BMI Δ change					−3.9 (2.2)	−4.3 (3.0)	−4.4 (2.1)	−2.9 (1.3)	−9.8 (4.7)	−12.2 (4.0)	−11.28 (4.5)	−6.1 (3.0)
Weight (kg)	111.9 (20.4)	115.0 (27.3)	113.1 (19.8)	107.2 (13.0)	102.3 (18.8)	104.6 (24.0)	103.2 (21.0)	98.9 (11.7)	86.0 (19.8)	83.0 (27.0)	82.2 (13.6)	92.7 (15.3)
Weight Δ change					−9.6 (5.4)	−10.4 (7.4)	−9.9 (5.2)	−8.4 (2.8)	−25.9 (11.9)	−32.0 (8.0)	−30.9 (12.6)	−14.6 (7.1)
ICP (cmCSF)	35.6 (4.5)	35.6 (5.6)	35.6 (4.7)	35.5 (3.6)	26.9 (8.1)	23.9 (6.8)	29.2 (12.2)	28.1 (3.8)	22.7 (5.4)	19.6 (4.4)	22.1 (6.1)	26.9 (3.1)
ICP Δ change					−9.0 (8.11)	−12.7 (10.5)	−6.4 (8.8)	−7.3 (1.3)	−12.9 (7.2)	−16.1 (8.4)	−13.5 (8.0)	−7.4 (3.9)

Baseline, 2 weeks post-surgery and 12-month time points for grouped and split by surgery types (RYGB, sleeve and band).

Data presented as mean ± SD.

*n*, number.

### Gut neuropeptide meal-stimulated changes

**Figure 2:**
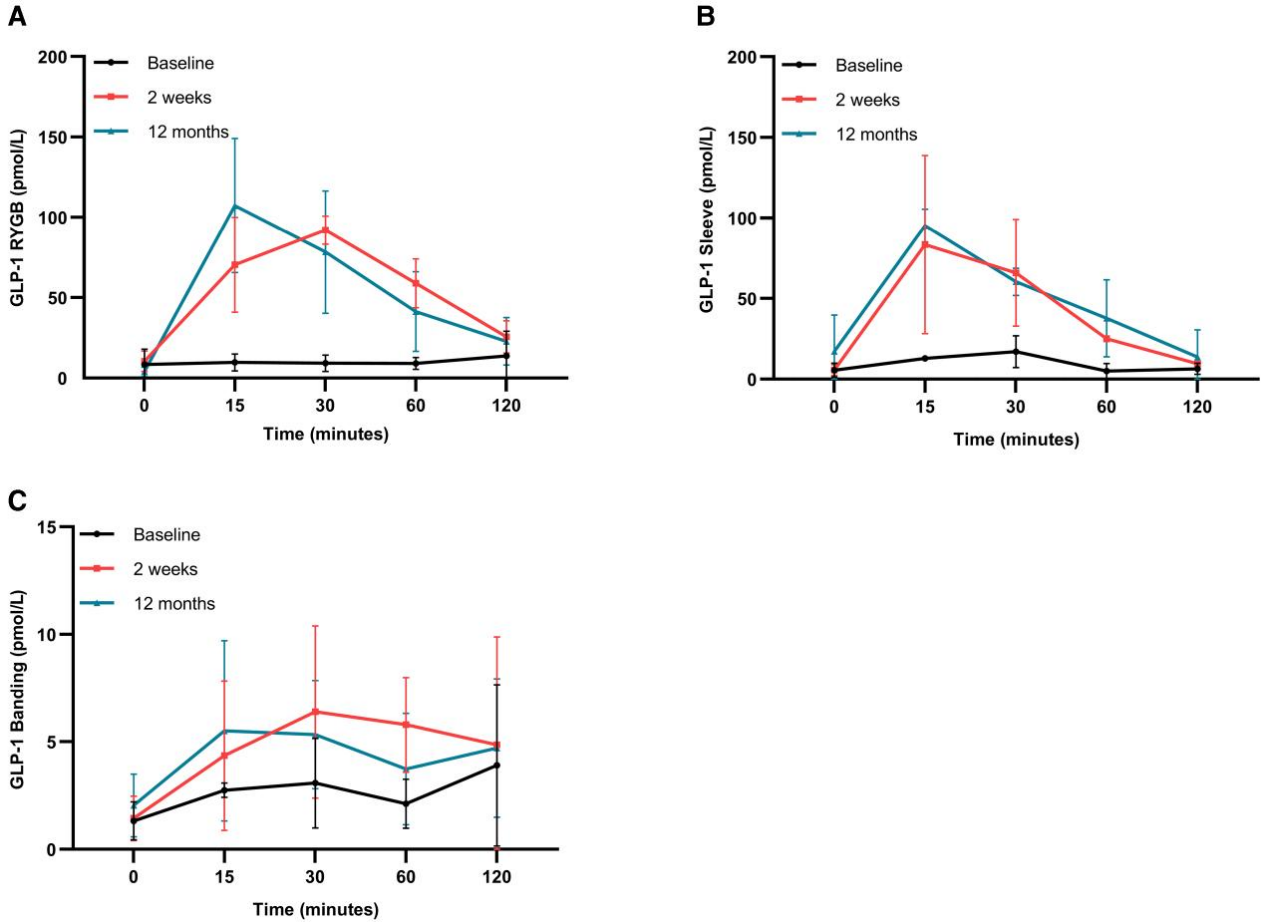
**GLP-1 gut hormone responses in all surgical cohorts at all time points**. Total AUC dynamics of GLP-1 RYGB (baseline total area: 1240 ± 490.6; 2 weeks total area: 6623 ± 688.4, +434%; 12 months total area: 5947 ± 1222, +380%) (*n* = 7) (**A**); gastric sleeve (baseline total area: 1045 ± 253.1; 2 weeks total area: 4194 ± 810.8, +301%; 12 months total area: 5029 ± 973.3, +381%) (*n* = 3) (**B**); gastric banding (baseline total area: 332.7 ± 124.0; 2 weeks total area: 626.4 ± 184.8, +88%; 12 months total area: 527.3 ± 143.9, +59%) (*n* = 5) (**C**) following a meal stimulation at 0 to 120 min as total area ± standard error, and percentage change over baseline. Only descriptive analysis was performed on this figure. No formal statistical testing was performed. Total area units = (pmol/L × minutes).

GLP-1, GIP and ghrelin were assessed over the meal stimulation protocol. In the RYGB and sleeve cohorts, the meal-stimulated GLP-1 area AUC_0 to 120_ profile showed a marked increase following surgery (both at 2 weeks and 12 months) compared to prior to surgery, with greater increases in the RYGB versus sleeve groups, in keeping with the literature^42,43^ (Fig. 2, RYGB (A) and gastric sleeve (B)). There was a greater increase in the meal-stimulated GLP-1 AUC_0 to 120_ profile following RYGB (434%) (*n* = 7) compared to sleeve (301%) (*n* = 3) at 2 weeks post-surgery. The increase was similar at 12 months between the two surgical groups (RYGB: 380% and sleeve: 381%). As expected, those undergoing gastric banding (*n* = 5) did not show a differential meal-stimulated GLP-1 AUC_0 to 120_ profile following surgery^43,44^ (Fig. 2C). Ghrelin showed no change over the entire meal-stimulated AUC_0 to 120_ profile (Supplementary Fig. 2A–C) in all surgery types. We would have expected to see a small decrease in ghrelin in the sleeve cohort due to the removal of the fundus of the stomach where most ghrelin-producing cells are located.^45^ However, this result may be an anomaly due to low sample numbers. There was also no significant change in GIP for all surgery types over the entire meal-stimulated AUC_0 to 120_ profile (Supplementary Fig. 2D–F), which is as expected and has been previously published.^43,46^

Small sample sizes confounded our ability to evaluate the relationship between GLP-1 and ICP. There was a significant negative correlation (*P* = 0.016, *r* = −0.608) between GLP-1 AUC_0 to 120_ and absolute ICP at 12 months in those undergoing bariatric surgery (*n* = 15). Correlations were not observed at 2 weeks or for the change between baseline and 2 weeks.

### Baseline meal-stimulated metabolite changes

**Figure 3:**
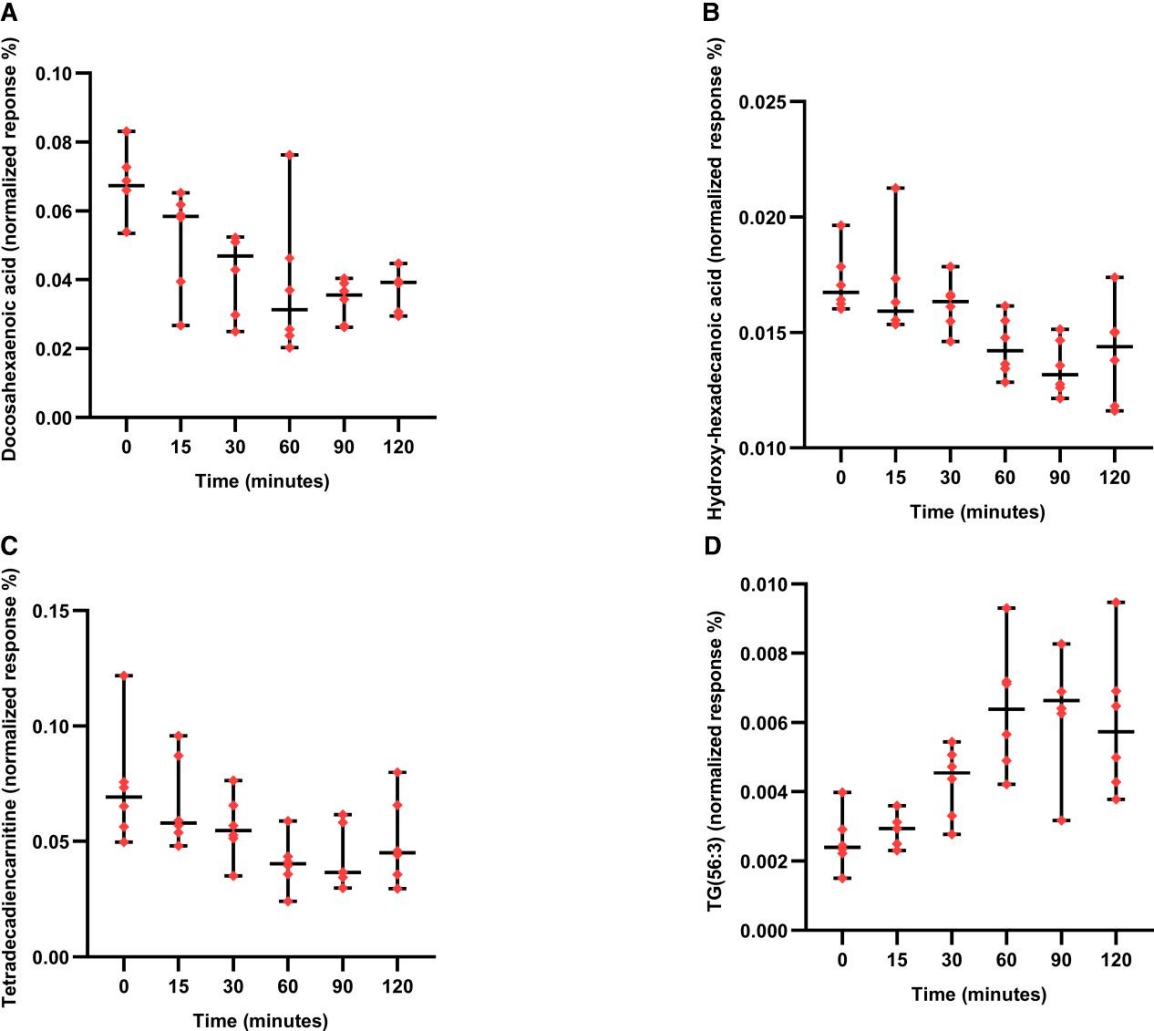
**Box and whisker plots demonstrate changes over time for specific lipid classes**. Total AUC as a normalized response % of docosahexaenoic acid (Total area: 4.943 ± 0.5455) (**A**); hydroxy-hexadecanoic acid (Total area: 1.794 ± 0.05924) (**B**); tetradecadiencarnitine (Total area: 6.072 ± 0.5966) (**C**); TG (56:3) (Total area: 0.6301 ± 0.6480) (**D**) at each time point (0, 15, 30, 60, 90 and 120 min) at baseline which is composed of timepoint matched IIH subjects prior to surgery (*n* = 6) as total area ± standard error. A one-way repeated measures ANOVA was applied with correction applied for multiple testing (Benjamini–Hochberg method) with a *q*-value < 0.05 to find the significant metabolite expression from the samples. Only descriptive analysis was performed on this figure. No formal statistical testing was performed.

The dynamic changes in metabolites, over the meal stimulation test, were initially evaluated prior to bariatric surgery for meal stimulation timepoint matched patients in the IIH cohort (Supplementary Table 2). We observed dynamic changes in 150 metabolites over the course of the meal stimulation test. Perturbations of metabolites were noted over a period of 120 min along with alterations in lipids [acyl amino acids (eight metabolites), acyl carnitines (27 metabolites), fatty acids (13 metabolites) and oxidized fatty acids (14 metabolites)]. Examples of the dynamic meal-stimulated changes for acyl carnitines, fatty acids, oxidized fatty acids and triacylglycerides are shown in Fig. 3.

We observed dynamic changes in vitamin A, D and E metabolites as well as nicotinamide, pantothenic acid, folic acid and iodine metabolism during the meal stimulation. It is likely that changes in these metabolites were influenced by the composition of the meal ingested (see Supplementary Table 1 for the full composition). These metabolites have not been further investigated and are not reported in Supplementary Table 2.

### Early (2 weeks) and late (12 months) meal-stimulated metabolite changes following bariatric surgery

**Figure 4:**
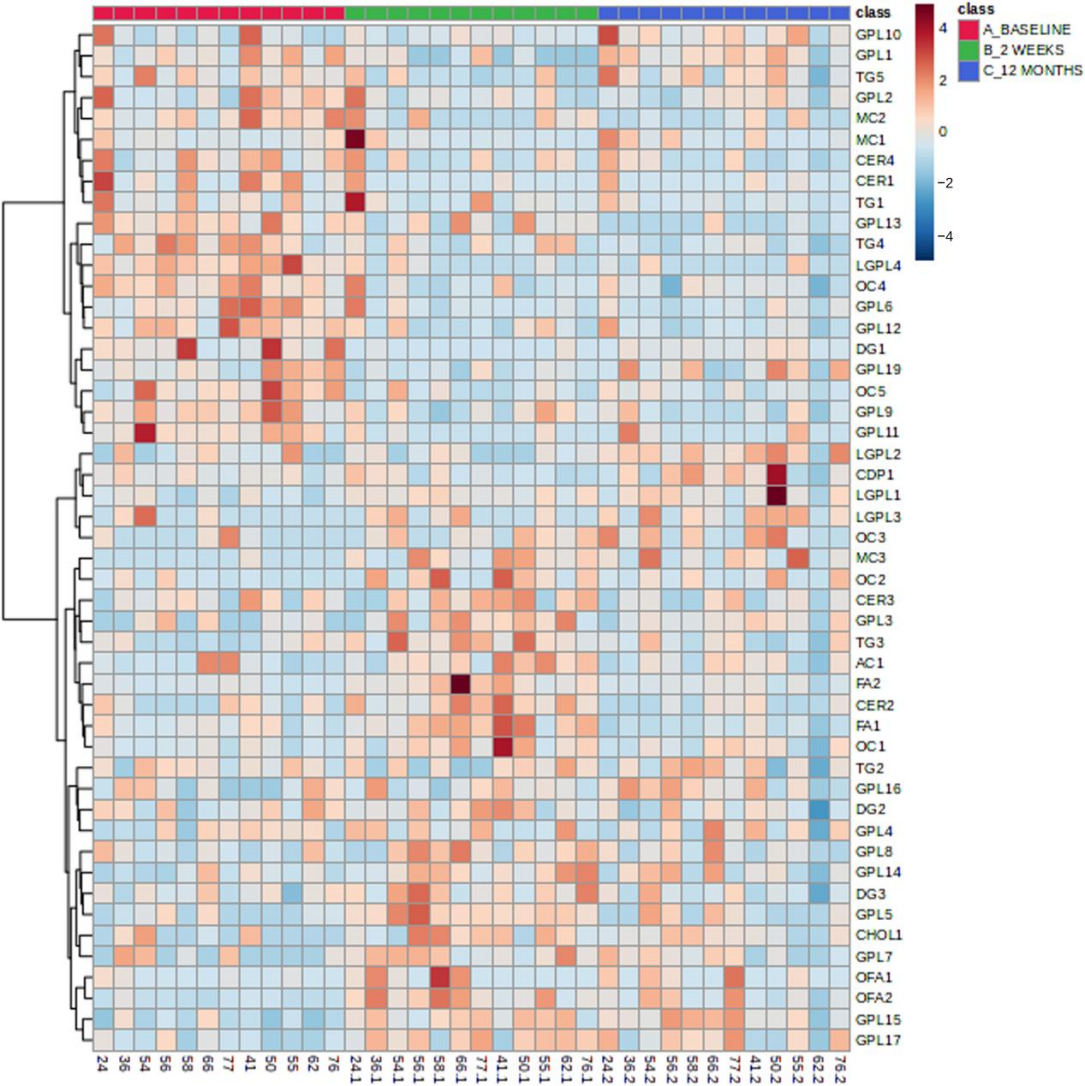
**Hierarchical clustering heatmap visualizing data for all IIH subjects (bypass and sleeve)**. Hierarchical Clustering Heatmaps were constructed in MetaboAnalyst using the following parameters: features (autoscaled), distance measure (Euclidean), clustering method (Ward) and samples (not clustered). A subset of lipids with *q* < 0.05 was chosen to visualize trends in the data. Baseline (magenta), 2 weeks post-surgery (green) and 12 months post-surgery (blue). All statistically significant metabolites for the comparison of baseline versus 2 weeks or baseline versus 12 months are included. Blue are low abundances and red are high abundances (*n* = 12). AC, acyl carnitine; GPL, glycerophospholipid; TG, triacylglyceride; MC, mixed class; CER, ceramide; LGPL, lysoglycerophospholipid; OC, other class; DG, diacyglyceride; CDP, glycerol; FA, fatty acid; OFA, oxidized fatty acid.

We next compared the alteration in dynamic meal-stimulated metabolites that occurred early (2 weeks; Supplementary Table 3—phenotype results) and late (12 months; Supplementary Table 4—phenotype results) after bariatric surgery in comparison to the pre-surgical meal stimulation test for all patients independent of surgery type. There were a number of similarities in metabolite changes observed at 2 weeks and 12 months post-surgery compared to pre-surgery with glycerophospholipids being the lipid class showing the most changes at both time points. Additionally, one cholesterol esters and 4-trimethylaminobutanoate were statistically significantly altered (for the direction of change, see Supplementary Tables 3 and 4) both early and late after bariatric surgery. A small number of ceramides, diacylglycerides, fatty acids as well as cholesterol sulphate were statistically significantly altered at 2 weeks but not 12 months after bariatric surgery. A hierarchical clustering heatmap visualization was used to display these differences in metabolite expression (Fig. 4).

### The impact of bariatric surgery type on early changes in dynamic meal-stimulated metabolites

We evaluated the dynamic meal-stimulated metabolite changes occurring early (2 weeks) post-surgery in comparison to pre-surgery dependent on the type of bariatric procedure [Supplementary Tables 5 (RYGB—phenotype results) and 6 (sleeve—phenotype results)]. We observed metabolite changes resulting from both types of bariatric surgery [26 and 41 metabolites were statistically significantly altered (for the direction of change, see Supplementary Tables 5 and 6) following RYGB and sleeve interventions, respectively]. Both surgical interventions resulted in statistically significant metabolite changes in glycerophospholipids; 9 of 26 and 25 of 41 for RYGB and sleeve, respectively.

We further investigated the differences between RYGB and sleeve surgery at 2 weeks post-surgery via a direct statistical comparison (RYGB at 2 weeks compared to sleeve at 2 weeks; Supplementary Table 7—phenotype results). We found that there are nine metabolites which are statistically significant when comparing RYGB at 2 weeks and sleeve at 2 weeks and five of the nine metabolites were (lyso)glycerophospholipids. Additionally, there were six metabolite changes in common at 2 weeks post-surgery for the RYGB and sleeve cohorts compared to baseline and we would suggest that these are less likely to be relevant to the increased ICP reduction in the RYGB cohort.

**Figure 5:**
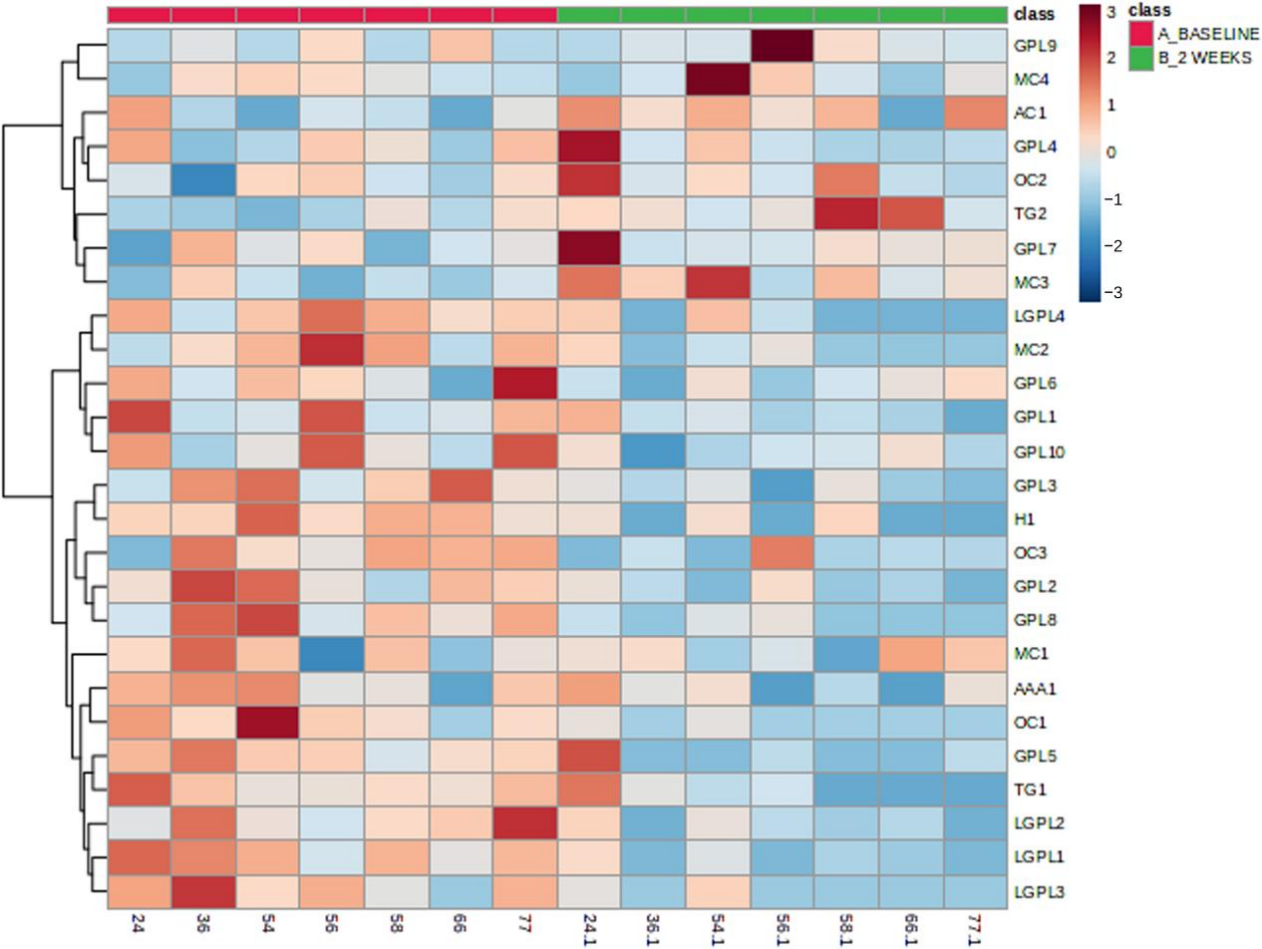
**Hierarchical clustering heatmap visualizing data for all bypass subjects**. Hierarchical Clustering Heatmaps were constructed in MetaboAnalyst using the following parameters: features (autoscaled), distance measure (Euclidean), clustering method (Ward) and samples (not clustered). A subset of lipids with *q* < 0.05 was chosen to visualize trends in the data. Baseline (red) and 2 weeks post-surgery (green). All statistically significant metabolites for the comparison of baseline versus 2 weeks are included. Blue are low abundances and red are high abundances. Bypass (*n* = 7). AC, acyl carnitine; GPL, glycerophospholipid; TG, triacylglyceride; MC, mixed class; LGPL, lysoglycerophospholipid; OC, other class; H, haeme; AAA, acyl amino acid; CER, ceramide; OFA, oxidized fatty acid.

**Figure 6:**
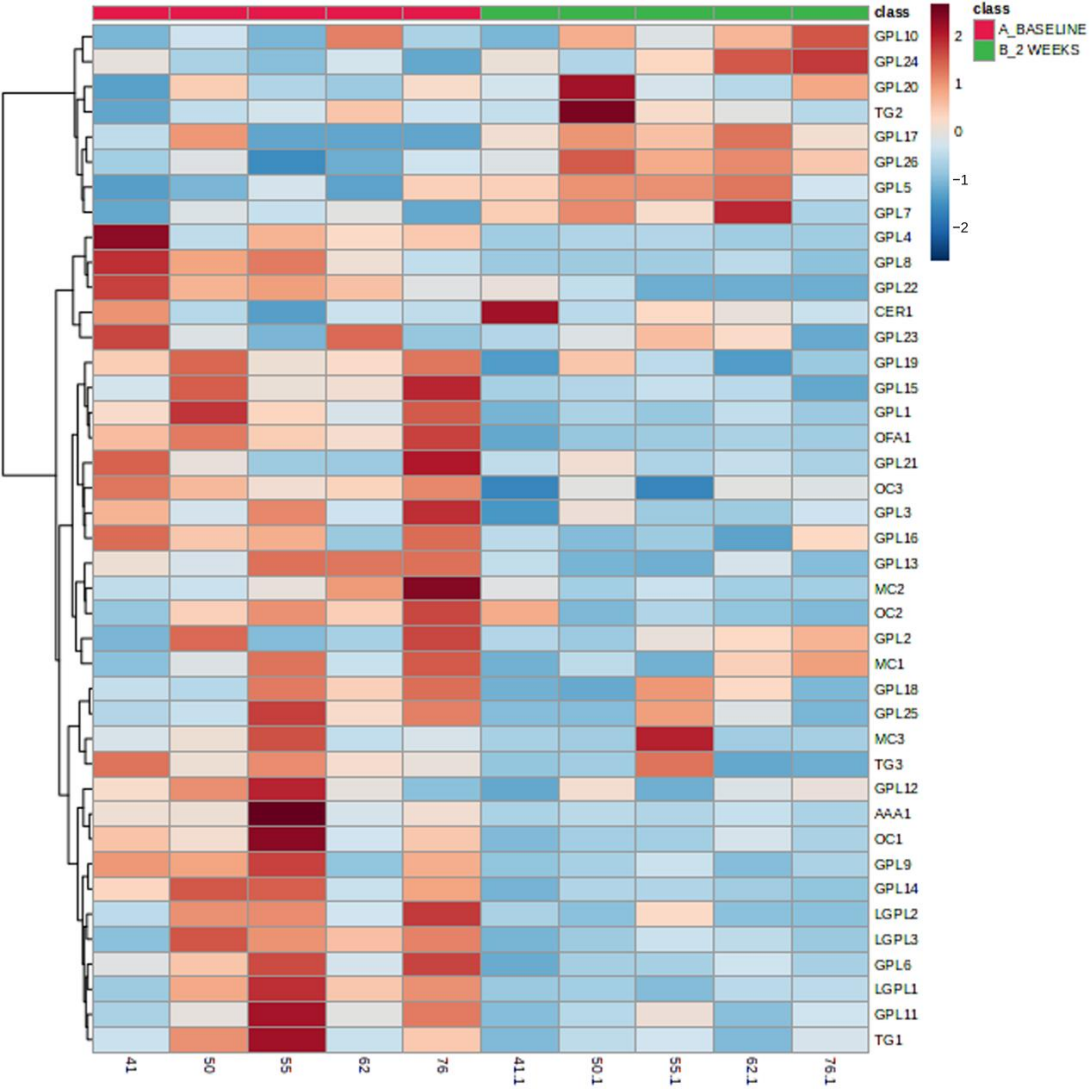
**Hierarchical clustering heatmap visualizing data for all sleeve subjects**. Hierarchical Clustering Heatmaps were constructed in MetaboAnalyst using the following parameters: features (autoscaled), distance measure (Euclidean), clustering method (Ward) and samples (not clustered). A subset of lipids with *q* < 0.05 was chosen to visualize trends in the data. Baseline (red) and 2 weeks post-surgery (green). All statistically significant metabolites for the comparison of baseline versus 2 weeks are included. Blue are low abundances and red are high abundances. Sleeve (*n* = 5). AC, acyl carnitine; GPL, glycerophospholipid; TG, triacylglyceride; MC, mixed class; LGPL, lysoglycerophospholipid; OC, other class; H, haeme; AAA, acyl amino acid; CER, ceramide; OFA, oxidized fatty acid.

Glycerophospholipids and lysoglycerophospholipids show a general decrease in relative concentration at 2 weeks compared to baseline for both RYGB and sleeve groups. However, many more glycerophospholipids were perturbed in sleeve patients (25) compared to RYGB patients (9) and the magnitude of change was greater in many of these lipids in sleeve patients compared to RYGB patients (Supplementary Tables 5 and 6; Fig. 4). A hierarchical clustering heatmap visualization was used to display expression of these metabolites between RYBG (Fig. 5) and sleeve (Fig. 6) surgery cohorts.

### The impact of bariatric surgery type on late changes in dynamic meal-stimulated metabolites

We next investigated whether the dynamic metabolite changes observed during a meal stimulation test late (12 months) post-surgery in comparison to pre-surgery were dependent on the type of bariatric procedure [Supplementary Tables 8 (RYGB—phenotype results) and 9 (sleeve—phenotype results)]. Fifty-five and 11 metabolites were statistically different from pre-surgery to 12 months post-surgery for RYGB and sleeve, respectively. For RYGB, 21 of 55 statistically significant metabolites were glycerophospholipids, and for sleeve, 6 of 11 metabolites were glycerophospholipids.

We further investigated the differences between RYGB and sleeve surgery at 12 months post-surgery via a direct statistical comparison (RYGB at 12 months compared to sleeve at 12 months). Thirteen metabolites were statistically significant (Supplementary Table 10).

### Relationship between intracranial pressure and metabolites

Next, we investigated the relationship between ICP, a measure of IIH disease activity, and metabolites. At 2 weeks post-bariatric surgery, we evaluated the fasted metabolites (those identified prior to the meal stimulation test) and their relationship to ICP. Seventy-five metabolites were correlated with ICP among all of those participants undergoing bariatric surgery (Supplementary Table 11; the abundance of 33 metabolites were negatively correlated to ICP and the abundance of 42 metabolites were positively correlated to ICP). Of these, we noted that two were ceramides, seven were glycerophospholipids (six of seven were positively correlated with ICP), seven were lysoglycerophospholipids (six of seven were positively correlated with ICP) and acetate. We then went on to evaluate the metabolites correlating with ICP at 2 weeks post-surgery in the RYGB group alone. A total of 147 metabolite correlations were observed (Supplementary Table 12; the abundance of 57 metabolites were negatively correlated to ICP and the abundance of 90 metabolites were positively correlated to ICP). Correlations in 17 ceramides (12 were positively correlated with ICP), 32 glycerophospholipids (21 were positively correlated with ICP) and 10 lysoglycerophospholipids (7 were negatively correlated with ICP) were observed, suggesting a relationship to ICP for these lipid classes. In addition, 130 metabolites were identified as correlating with ICP in the RYGB group alone but not in the whole cohort (Supplementary Table 12B), with LysoPS (P-20:0) metabolite found to be statistically significant in both the changes in metabolites (baseline—2 weeks) and correlated changes in ICP (baseline—2 weeks). These may also be of biological relevance in explaining the disproportionate reduction in ICP in the RYGB group compared to sleeve.

The smaller number of correlated ceramides and glycerophospholipids observed in the whole IIH cohort (RYGB and sleeve combined) compared to the RYGB group alone suggest that these two lipid classes are meaningfully associated with ICP in the RYGB group (as these correlations are lost when the sleeve group is combined with the RYGB group). Hence, ceramides and glycerophospholipids may be relevant to the disproportionate reduction in ICP observed in the RYGB group at 2 weeks post-surgery.

We also evaluated the relationship between changes in metabolites (between baseline and 2 weeks post-surgery) and changes in ICP in the whole bariatric cohort over this time period. Around 152 metabolites were correlated (Supplementary Table 13; the change in the abundance of 95 metabolites was negatively correlated to the change in ICP and the change in the abundance of 57 metabolites was positively correlated to the change in ICP) of which 7 were ceramides, 34 glycerophospholipids and 22 lysoglycerophospholipids. However, in the RYGB cohort alone, 160 metabolite changes correlated with change in ICP (the change in the abundance of 96 metabolites was negatively correlated to the change in ICP and the change in the abundance of 64 metabolites was positively correlated to the change in ICP). Of these, 11 were ceramides, 38 glycerophospholipids and 15 lysoglycerophospholipids (Supplementary Table 14). Both glycerophospholipids and lysoglycerophospholipids were negatively correlated with ICP change and within the class of ceramide metabolites, some ceramides showed positive and some showed negative correlations with ICP change. This suggests that these three lipid classes are associated with changes in ICP and therefore potentially important in understanding the mechanisms driving reduction in ICP following weight loss post-bariatric surgery. Additionally, those correlations predominantly associated with RYGB may be relevant in explaining the exaggerated reduction in ICP amongst the RYGB cohort.

## Discussion

This study sought to explore the potential mechanisms by which weight loss could exert a therapeutic effect in IIH and lower ICP. We observed rapid improvements in ICP after bariatric surgery at 2 weeks, particularly in the RYGB cohort, and hence sought to additionally explore mechanisms driving these early changes in ICP. Our data suggests that changes in GLP-1 levels and specific lipid metabolites may contribute to the reduction in ICP observed.

### Role of gut neuropeptides

A very early reduction in ICP was identified at 2 weeks post-bariatric surgery. We noted that this was particularly marked among the group undergoing RYGB surgery (almost a 2-fold greater reduction in ICP compared to the gastric sleeve cohort) despite similar reductions in weight. This was akin to what has been observed in T2D where early glycaemic control at 2 weeks post-RYGB surgery is noted in line with changes in gut neuropeptides.^32^ In our cohort, at 2 weeks post-bariatric surgery the meal-stimulated secretion of ghrelin and GIP showed no change, while meal-stimulated GLP-1 secretion altered particularly in those undergoing RYGB, as has been noted in other diseases.^43,45,47^ The greater increases in GLP-1 associated with RYGB compared to gastric sleeve patients hve been previously linked to elevated nutrient-stimulated circulating levels of GLP-1 due to increased stimulation of L-cells.^43^

This was a small study which limits the interpretation of the results; however, our data did suggest an association between a reduction in ICP and an increase in meal-stimulated GLP-1 levels. This is in keeping with existing data demonstrating the importance of GLP-1 in ICP regulation in both pre-clinical and clinical data.^27,29^ The observed exaggerated reduction in ICP in the RYGB cohort could be driven by changes in GLP-1 levels following surgery, but in this small cohort, further confirmatory studies are needed. GLP-1 agonist infusions have been evaluated due to their potential to replicate changes occurring after RYGB.^48^ We speculate that GLP-1 agonists could have therapeutic potential in IIH in lieu of RYGB.

### Lipid metabolites and ICP reduction

Comparing the untargeted metabolomics after meal stimulation testing, before and after bariatric surgery, the predominant metabolite classes to show significant dynamic changes were ceramides, glycerophospholipids and lysoglycerophospholipids. Furthermore, these lipid metabolites were associated with changes in ICP. We cannot discern a causal relationship from this data; however, we would suggest that the results indicate that these lipid metabolites may have a mechanistic role in ICP reduction following weight loss. In support of this, it is known that obesity and recent weight gain are known risk factors for IIH and significant weight loss has been shown to be a disease-modifying treatment of IIH.^12,13,49^ Back translation to evaluate ceramides, glycerophospholipids and lysoglycerophospholipids in animal models of CSF dynamics would be of interest.

### Differential metabolite changes by surgical type

The changes in the lipid metabolites (ceramides, glycerophospholipids and lysoglycerophospholipids) were even more pronounced in those undergoing RYGB compared to sleeve (both at 2 weeks and 12 months). Additionally, there were a larger number of correlations between lipid metabolites and ICP in the RYGB cohort. Hence, changes in lipid metabolites appear to be relevant to the reduction in ICP and this relationship is amplified amongst the RYGB cohort suggesting their role is partially independent of weight loss. This is potentially relevant to understanding the exaggerated reduction of ICP in the RYGB cohort. We also note that changes in acetate correlated with ICP in this study. This is in keeping with elevated acetate levels identified in IIH compared to control subjects previously observed using nuclear magnetic resonance spectroscopy metabolite analysis.^22^

### Lipid metabolites, obesity and weight loss

The mechanisms by which ceramides, glycerophospholipids and lysoglycerophospholipids may be involved in ICP reduction following weight loss is not known. These lipid metabolite classes have been previously associated with obesity and metabolic syndrome.^50-53^ Changes in these metabolites have been noted following bariatric surgery^32,50-53^ and after weight loss following lifestyle interventions,^54-58^ hence are not specific to bariatric surgery but more a marker of weight loss independent of the mode of weight loss.

In IIH, HILIC-based UHPLC-MS has identified that multiple ceramides, fatty acids, glycerophospholipids and lysoglycerophospholipids are altered in association with ICP.^59^ RYBG has been previously shown to perturb ceramide levels.^32^ Ceramide dysregulation has been implicated in insulin resistance and CVD risk in other diseases^9,10,60^ and may be of relevance in IIH where an increased risk of CVD and insulin resistance has been observed.^9^

Ceramides belong to the sphingolipid family and are produced from the breakdown of membrane sphingomyelin by sphingomyelinase.^61^ The brain is the organ with the highest sphingolipid content in the body.^62^ Additionally, sphingomyelins are abundant in the myelin layer in the optic nerve sheath.^63,64^ The optic nerve sheath is dilated in IIH as ICP rises.^65^ We hypothesize that dilation of the optic nerve sheath could lead to mechanical stress and sphingomyelin breakdown and production of ceramide. Hence, ceramide could reflect a biomarker of elevated ICP in IIH. It is interesting to note that changes in the brain’s lipid levels, including glycerophospholipids,^66^ are associated with other pathogenic processes including neuroinflammatory diseases such as Alzheimer’s disease and Parkinson’s disease.^67-69^ Future work to explore the impact of lipid metabolites on ICP regulation would be of interest.

## Limitations

There are some limitations to the reported study which should be considered. Due to the rarity of the disease, there were relatively small numbers of participants in the surgery cohorts. However, this was the first study to explore the changes of metabolites following bariatric surgery in IIH patients and this sample size is larger compared to other IIH studies. In addition, the small sample size may have limited the ability to discern charges in some metabolites and gut neuropeptides. For example, existing literature suggests that ghrelin alters after sleeve gastrectomy, but in this small study, we did not observe this change. Untargeted UHPLC-MS metabolomics is not able to quantify metabolite changes, but this would be of future interest. UHPLC-MS does, however, have the advantage of being untargeted, and hence, our findings reflect a broad discovery-based approach. We have not evaluated the functional significance of our findings currently, but this study guides future research work. It is not possible to say if changes in lipid metabolites are a cause or consequence of ICP changes until further functional evaluations are conducted. There was no non-IIH cohort, in which meal-stimulated samples were collected (as this work has previously been conducted).^32^ However, the aim of this study was not to determine if metabolite changes after bariatric surgery were unique to IIH, but to determine how metabolite changes in IIH related to changes in ICP. Metabolites were sought in plasma; however, it would be a future interest to analyse the changes in metabolite signatures in the CSF following bariatric surgery.

Some metabolites are identified based on the comparison of retention time and/or MS/MS data to data collected for authentic chemical standards, though other metabolites are annotated without comparison to chemical standards. For this reason, we have applied pathway enrichment analysis to reduce (but not fully eliminate) the probability of false positive conclusions. For example, if eight metabolites are statistically significant and present in a single pathway, then we have more confidence that this is a biologically valid conclusion compared to deriving biological conclusions from a single statistically significant metabolite without applying pathway enrichment analysis.

## Conclusion

**Figure 7:**
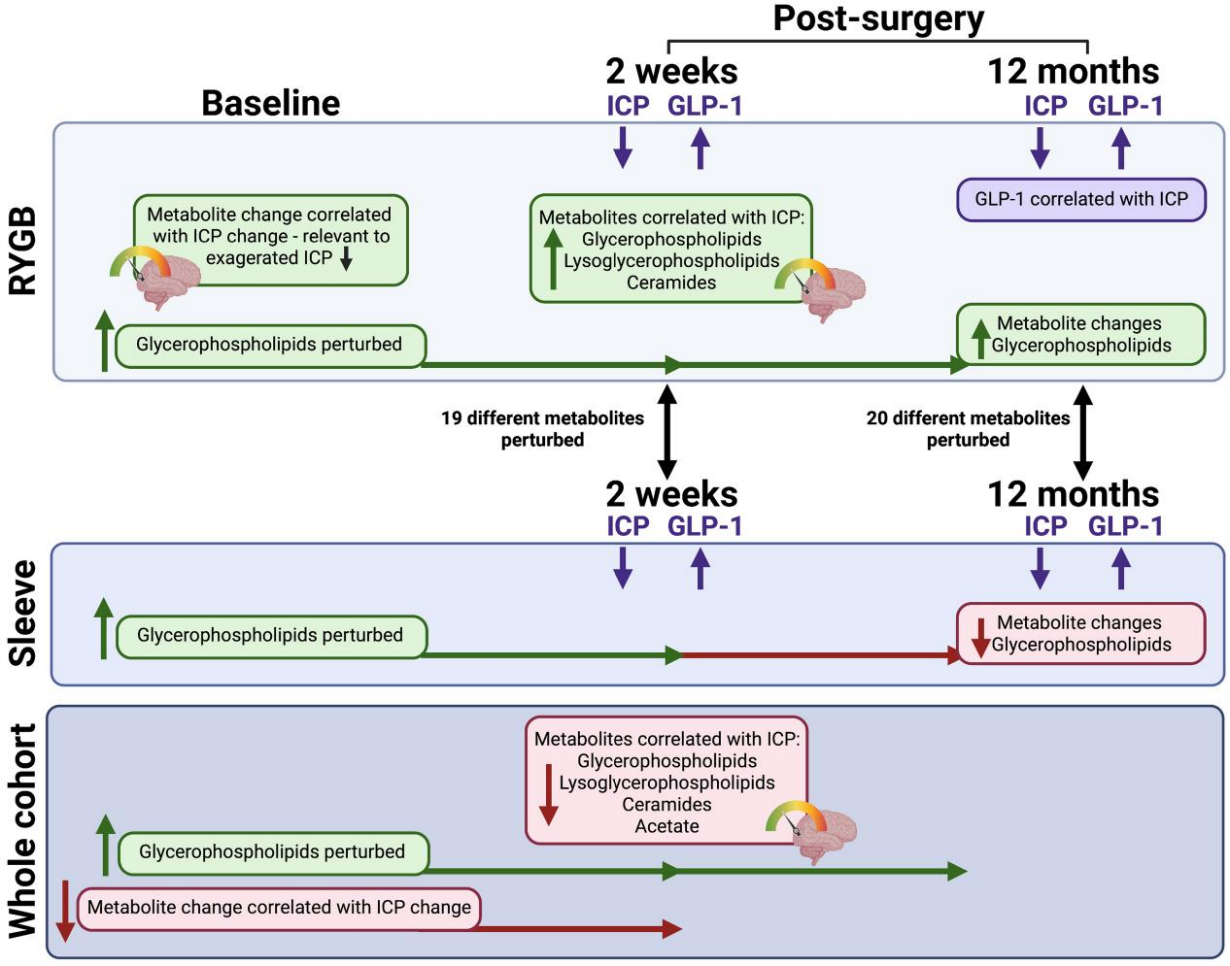
**Infographic**. In IIH, novel changes were noticed in lipid metabolites (glycerphospholipids, lysoglycerphospholipids and ceramides) and meal-stimulated GLP-1 levels, in patients following RYGB surgery, which were associated with changes in ICP. Nineteen and 20 different metabolites were perturbed between the RYGB and sleeve cohorts at 2 weeks and 12 months post-surgery, respectively.

Reduction in weight, driven by bariatric surgery, both early (2 weeks) and late (12 months), was associated with novel changes in lipid metabolism (notably ceramides, glycerophospholipids and lysoglycerphospholipids) which correlated with changes in ICP. We observed rapid improvements in ICP at 2 weeks post-bariatric surgery, particularly in the RYGB group, despite similar degrees of weight loss in the other surgical types. At 2 weeks post-surgery, changes in lipid metabolism and GLP-1 levels were of greater magnitude in the RYGB group, potentially indicating their importance in driving the exaggerated ICP reduction in this surgical type. Ceramides, glycerophospholipids and lysoglycerphospholipids were strongly associated with changes in ICP at 2 weeks post-surgery in the RYGB cohort (Fig. 7). The mechanisms by which changes in lipid metabolites influence ICP are yet to be explored. We suggest that these novel perturbations in lipid metabolism and GLP-1 secretion are mechanistically important in driving reduction in ICP following weight loss in patients with IIH. However, the small sample size precludes firm conclusions being drawn. Therapeutic targeting, however, of these pathways, for example with GLP-1 agonist administration, could represent a therapeutic strategy.

## Supplementary Material

fcad272_Supplementary_Data

## Data Availability

The authors confirm that the data supporting the findings of this study are available within the article and its Supplementary material.

## References

[1] Mollan SP , Sinclair AJ. Outcomes measures in idiopathic intracranial hypertension. Expert Rev Neurother. 2021;21(6):687–700. 34047224 10.1080/14737175.2021.1931127

[2] Thaller M , Homer V, Hyder Y, et al The idiopathic intracranial hypertension prospective cohort study: Evaluation of prognostic factors and outcomes. J Neurol. 2023;270(2):851–863. 36242625 10.1007/s00415-022-11402-6 PMC9886634

[3] Corbett JJ , Savino PJ, Thompson HS, et al Visual loss in pseudotumor cerebri. Follow-up of 57 patients from five to 41 years and a profile of 14 patients with permanent severe visual loss. Arch Neurol. 1982;39(8):461–474. 7103794 10.1001/archneur.1982.00510200003001

[4] Mollan SP , Grech O, Sinclair AJ. Headache attributed to idiopathic intracranial hypertension and persistent post-idiopathic intracranial hypertension headache: A narrative review. Headache. 2021;61(6):808–816. 34106464 10.1111/head.14125

[5] Mollan SP , Wakerley BR, Alimajstorovic Z, et al Intracranial pressure directly predicts headache morbidity in idiopathic intracranial hypertension. J Headache Pain. 2021;22(1):118. 34620087 10.1186/s10194-021-01321-8 PMC8499560

[6] Virdee J , Larcombe S, Vijay V, Sinclair AJ, Dayan M, Mollan SP. Reviewing the recent developments in idiopathic intracranial hypertension. Ophthalmol Ther. 2020;9(4):767–781. 32902722 10.1007/s40123-020-00296-0 PMC7708542

[7] Mollan SP , Tahrani AA, Sinclair AJ. The potentially modifiable risk factor in idiopathic intracranial hypertension: Body weight. Neurol Clin Pract. 2021;11(4):e504–e507. 34484948 10.1212/CPJ.0000000000001063 PMC8382420

[8] Mollan SP , Aguiar M, Evison F, Frew E, Sinclair AJ. The expanding burden of idiopathic intracranial hypertension. Eye (Lond). 2019;33(3):478–485. 30356129 10.1038/s41433-018-0238-5 PMC6460708

[9] Adderley NJ , Subramanian A, Nirantharakumar K, et al Association between idiopathic intracranial hypertension and risk of cardiovascular diseases in women in the United Kingdom. JAMA Neurol. 2019;76(9):1088–1098. 31282950 10.1001/jamaneurol.2019.1812 PMC6618853

[10] Westgate CS , Botfield HF, Alimajstorovic Z, et al Systemic and adipocyte transcriptional and metabolic dysregulation in idiopathic intracranial hypertension. JCI Insight. 2021;6(10). 10.1172/jci.insight.145346 PMC8262372 33848268

[11] Hornby C , Botfield H, O'Reilly MW, et al Evaluating the fat distribution in idiopathic intracranial hypertension using dual-energy X-ray absorptiometry scanning. Neuroophthalmology. 2018;42(2):99–104. 29563954 10.1080/01658107.2017.1334218 PMC5858863

[12] Mollan SP , Mitchell JL, Ottridge RS, et al Effectiveness of bariatric surgery vs community weight management intervention for the treatment of idiopathic intracranial hypertension: A randomized clinical trial. JAMA Neurol. 2021;78(6):678–686. 33900360 10.1001/jamaneurol.2021.0659 PMC8077040

[13] Sinclair AJ , Burdon MA, Nightingale PG, et al Low energy diet and intracranial pressure in women with idiopathic intracranial hypertension: Prospective cohort study. BMJ. 2010;341:c2701. 20610512 10.1136/bmj.c2701 PMC2898925

[14] Yiangou A , Mitchell JL, Nicholls M, et al Obstructive sleep apnoea in women with idiopathic intracranial hypertension: A sub-study of the idiopathic intracranial hypertension weight randomised controlled trial (IIH:WT). J Neurol. 2022;269(4):1945–1956. 34420064 10.1007/s00415-021-10700-9 PMC8940816

[15] Westgate CSJ , Markey K, Mitchell JL, et al Increased systemic and adipose 11β-HSD1 activity in idiopathic intracranial hypertension. Eur J Endocrinol. 2022;187(2):323–333. 35584002 10.1530/EJE-22-0108 PMC9346265

[16] O'Reilly MW , Westgate CS, Hornby C, et al A unique androgen excess signature in idiopathic intracranial hypertension is linked to cerebrospinal fluid dynamics. JCI Insight. 2019;4(6):e125348. 30753168 10.1172/jci.insight.125348 PMC6483000

[17] Hardy RS , Botfield H, Markey K, et al 11βHSD1 Inhibition with AZD4017 improves lipid profiles and lean muscle mass in idiopathic intracranial hypertension. J Clin Endocrinol Metab. 2021;106(1):174–187. 33098644 10.1210/clinem/dgaa766 PMC7765633

[18] Thaller M , Mytton J, Wakerley BR, Mollan SP, Sinclair AJ. Idiopathic intracranial hypertension: Evaluation of births and fertility through the Hospital Episode Statistics dataset. BJOG. 2022;129(12):2019–2027. 35620863 10.1111/1471-0528.17241 PMC9796176

[19] Thaller M , Wakerley BR, Abbott S, et al Managing idiopathic intracranial hypertension in pregnancy: Practical advice. Pract Neurol. 2022;22(4):295–300. 35450962 10.1136/practneurol-2021-003152 PMC9304112

[20] Grech O , Mollan SP, Wakerley BR, et al The role of metabolism in migraine pathophysiology and susceptibility. Life (Basel). 2021;11(5):415. 34062792 10.3390/life11050415 PMC8147354

[21] Grech O , Sassani M, Terwindt G, Lavery GG, Mollan SP, Sinclair AJ. Alterations in metabolic flux in migraine and the translational relevance. J Headache Pain. 2022;23(1):127. 36175833 10.1186/s10194-022-01494-w PMC9523955

[22] Grech O , Seneviratne SY, Alimajstorovic Z, et al Nuclear magnetic resonance spectroscopy metabolomics in idiopathic intracranial hypertension to identify markers of disease and headache. Neurology. 2022;99(16):e1702–e1714. 36240084 10.1212/WNL.0000000000201007 PMC9620805

[23] Vilsbøll T , Christensen M, Junker AE, Knop FK, Gluud LL. Effects of glucagon-like peptide-1 receptor agonists on weight loss: Systematic review and meta-analyses of randomised controlled trials. BMJ. 2012;344:d7771. 22236411 10.1136/bmj.d7771 PMC3256253

[24] Campbell JE , Drucker DJ. Pharmacology, physiology, and mechanisms of incretin hormone action. Cell Metab. 2013;17(6):819–837. 23684623 10.1016/j.cmet.2013.04.008

[25] Larsen PJ , Tang-Christensen M, Holst JJ, Orskov C. Distribution of glucagon-like peptide-1 and other preproglucagon-derived peptides in the rat hypothalamus and brainstem. Neuroscience. 1997;77(1):257–270. 9044391 10.1016/s0306-4522(96)00434-4

[26] Astrup A , Rössner S, Van Gaal L, et al Effects of liraglutide in the treatment of obesity: A randomised, double-blind, placebo-controlled study. Lancet. 2009;374(9701):1606–1616. 19853906 10.1016/S0140-6736(09)61375-1

[27] Botfield HF , Uldall MS, Westgate CSJ, et al A glucagon-like peptide-1 receptor agonist reduces intracranial pressure in a rat model of hydrocephalus. Sci Transl Med. 2017;9(404):eaan0972. 28835515 10.1126/scitranslmed.aan0972

[28] Ast J , Arvaniti A, Fine NHF, et al Super-resolution microscopy compatible fluorescent probes reveal endogenous glucagon-like peptide-1 receptor distribution and dynamics. Nat Commun. 2020;11(1):467. 31980626 10.1038/s41467-020-14309-w PMC6981144

[29] Mitchell JL , Lyons HS, Walker JK, et al The effect of GLP-1RA exenatide on idiopathic intracranial hypertension: Randomised clinical trial. Brain. 2023;146(5):1821–1830. 36907221 10.1093/brain/awad003 PMC10151178

[30] Mollan SP , Mitchell JL, Yiangou A, et al Association of amount of weight lost after bariatric surgery with intracranial pressure in women with idiopathic intracranial hypertension. Neurology. 2022;99(11):e1090–e1099. 35790425 10.1212/WNL.0000000000200839 PMC9536743

[31] Behary P , Tharakan G, Alexiadou K, et al Combined GLP-1, oxyntomodulin, and peptide YY improves body weight and glycemia in obesity and prediabetes/type 2 diabetes: A randomized, single-blinded, placebo-controlled study. Diabetes Care. 2019;42(8):1446–1453. 31177183 10.2337/dc19-0449

[32] Jones B , Sands C, Alexiadou K, et al The metabolomic effects of tripeptide gut hormone infusion compared to Roux-en-Y gastric bypass and caloric restriction. J Clin Endocrinol Metab. 2022;107(2):e767–e782. 34460933 10.1210/clinem/dgab608 PMC8764224

[33] Sudlow A , le Roux CW, Pournaras DJ. Review of multimodal treatment for type 2 diabetes: Combining metabolic surgery and pharmacotherapy. Ther Adv Endocrinol Metab. 2019;10:2042018819875407. 10.1177/2042018819875407 PMC6759694 31579501

[34] Abdeen G , le Roux CW. Mechanism underlying the weight loss and complications of Roux-en-Y gastric bypass. Review. Obes Surg. 2016;26(2):410–421. 26530712 10.1007/s11695-015-1945-7 PMC4709370

[35] Ottridge R , Mollan SP, Botfield H, et al Randomised controlled trial of bariatric surgery versus a community weight loss programme for the sustained treatment of idiopathic intracranial hypertension: The Idiopathic Intracranial Hypertension Weight Trial (IIH:WT) protocol. BMJ Open. 2017;7(9):e017426. 10.1136/bmjopen-2017-017426 PMC5623580 28963303

[36] Elliot L , Frew E, Mollan SP, et al Cost-effectiveness of bariatric surgery versus community weight management to treat obesity-related idiopathic intracranial hypertension: Evidence from a single-payer healthcare system. Surg Obes Relat Dis. 2021;17(7):1310–1316. 33952427 10.1016/j.soard.2021.03.020 PMC8241428

[37] Khoury JM , Donahue SP, Lavin PJ, Tsai JC. Comparison of 24-2 and 30-2 perimetry in glaucomatous and nonglaucomatous optic neuropathies. J Neuroophthalmol. 1999;19(2):100–108. 10380130

[38] Vijay V , Mollan SP, Mitchell JL, et al Using optical coherence tomography as a surrogate of measurements of intracranial pressure in idiopathic intracranial hypertension. JAMA Ophthalmol. 2020;138(12):1264–1271. 33090189 10.1001/jamaophthalmol.2020.4242 PMC7582233

[39] le Roux CW , Aylwin SJ, Batterham RL, et al Gut hormone profiles following bariatric surgery favor an anorectic state, facilitate weight loss, and improve metabolic parameters. Ann Surg. 2006;243(1):108–114. 16371744 10.1097/01.sla.0000183349.16877.84 PMC1449984

[40] McGlone ER , Malallah K, Cuenco J, et al Differential effects of bile acids on the postprandial secretion of gut hormones: A randomized crossover study. Am J Physiol Endocrinol Metab. 2021;320(4):E671–E679. 33459181 10.1152/ajpendo.00580.2020

[41] Pang Z , Chong J, Zhou G, et al Metaboanalyst 5.0: Narrowing the gap between raw spectra and functional insights. Nucleic Acids Res. 2021;49(W1):W388–WW96. 34019663 10.1093/nar/gkab382 PMC8265181

[42] Dirksen C , Jørgensen NB, Bojsen-Møller KN, et al Gut hormones, early dumping and resting energy expenditure in patients with good and poor weight loss response after Roux-en-Y gastric bypass. Int J Obes (Lond). 2013;37(11):1452–1459. 23419600 10.1038/ijo.2013.15

[43] Pucci A , Batterham RL. Mechanisms underlying the weight loss effects of RYGB and SG: Similar, yet different. J Endocrinol Invest. 2019;42(2):117–128. 29730732 10.1007/s40618-018-0892-2 PMC6394763

[44] Cummings DE , Weigle DS, Frayo RS, et al Plasma ghrelin levels after diet-induced weight loss or gastric bypass surgery. N Engl J Med. 2002;346(21):1623–1630. 12023994 10.1056/NEJMoa012908

[45] Yousseif A , Emmanuel J, Karra E, et al Differential effects of laparoscopic sleeve gastrectomy and laparoscopic gastric bypass on appetite, circulating acyl-ghrelin, peptide YY3-36 and active GLP-1 levels in non-diabetic humans. Obes Surg. 2014;24(2):241–252. 23996294 10.1007/s11695-013-1066-0 PMC3890046

[46] Alexiadou K , Cuenco J, Howard J, et al Proglucagon peptide secretion profiles in type 2 diabetes before and after bariatric surgery: 1-year prospective study. BMJ Open Diabetes Res Care. 2020;8(1):e001076. 10.1136/bmjdrc-2019-001076 PMC7103850 32209584

[47] Dar MS , Chapman WH III, Pender JR, et al GLP-1 response to a mixed meal: What happens 10 years after Roux-en-Y gastric bypass (RYGB)? Obes Surg. 2012;22(7):1077–1083. 22419108 10.1007/s11695-012-0624-1

[48] Hutch CR , Sandoval D. The role of GLP-1 in the metabolic success of bariatric surgery. Endocrinology. 2017;158(12):4139–4151. 29040429 10.1210/en.2017-00564 PMC5711387

[49] Mollan SP , Davies B, Silver NC, et al Idiopathic intracranial hypertension: Consensus guidelines on management. J Neurol Neurosurg Psychiatry. 2018;89(10):1088–1100. 29903905 10.1136/jnnp-2017-317440 PMC6166610

[50] Kang M , Yoo HJ, Kim M, Kim M, Lee JH. Metabolomics identifies increases in the acylcarnitine profiles in the plasma of overweight subjects in response to mild weight loss: A randomized, controlled design study. Lipids Health Dis. 2018;17(1):237. 30322392 10.1186/s12944-018-0887-1 PMC6190541

[51] Perng W , Rifas-Shiman SL, Sordillo J, Hivert MF, Oken E. Metabolomic profiles of overweight/obesity phenotypes during adolescence: A cross-sectional study in Project Viva. Obesity (Silver Spring). 2020;28(2):379–387. 31876390 10.1002/oby.22694 PMC6980913

[52] Monnerie S , Comte B, Ziegler D, et al Metabolomic and lipidomic signatures of metabolic syndrome and its physiological components in adults: A systematic review. Sci Rep. 2020;10(1):669. 31959772 10.1038/s41598-019-56909-7 PMC6971076

[53] Yin X , Willinger CM, Keefe J, et al Lipidomic profiling identifies signatures of metabolic risk. EBioMedicine. 2020;51:102520. 31877415 10.1016/j.ebiom.2019.10.046 PMC6938899

[54] Schenk S , Horowitz JF. Acute exercise increases triglyceride synthesis in skeletal muscle and prevents fatty acid-induced insulin resistance. J Clin Invest. 2007;117(6):1690–1698. 17510709 10.1172/JCI30566 PMC1866251

[55] Dubé JJ , Amati F, Stefanovic-Racic M, Toledo FGS, Sauers SE, Goodpaster BH. Exercise-induced alterations in intramyocellular lipids and insulin resistance: The athlete's paradox revisited. Am J Physiol Endocrinol Metab. 2008;294(5):E882–E888. 18319352 10.1152/ajpendo.00769.2007 PMC3804891

[56] Dubé JJ , Amati F, Toledo FG, et al Effects of weight loss and exercise on insulin resistance, and intramyocellular triacylglycerol, diacylglycerol and ceramide. Diabetologia. 2011;54(5):1147–1156. 21327867 10.1007/s00125-011-2065-0 PMC3804898

[57] Andersson A , Sjödin A, Olsson R, Vessby B. Effects of physical exercise on phospholipid fatty acid composition in skeletal muscle. Am J Physiol. 1998;274(3):E432–E438. 9530125 10.1152/ajpendo.1998.274.3.E432

[58] San Martin R , Brandao CFC, Junqueira-Franco MVM, et al Untargeted lipidomic analysis of plasma from obese women submitted to combined physical exercise. Sci Rep. 2022;12(1):11541. 35798803 10.1038/s41598-022-15236-0 PMC9263166

[59] Alimajstorovic Z , Mollan SP, Grech O, et al Dysregulation of amino acid, lipid, and acylpyruvate metabolism in idiopathic intracranial hypertension: A non-targeted case control and longitudinal metabolomic study. J Proteome Res. 2022; 22(4):1127–1137 36534069 10.1021/acs.jproteome.2c00449 PMC10088035

[60] Field BC , Gordillo R, Scherer PE. The role of ceramides in diabetes and cardiovascular disease regulation of ceramides by adipokines. Front Endocrinol (Lausanne). 2020;11:569250. 33133017 10.3389/fendo.2020.569250 PMC7564167

[61] Lee JY , Jin HK, Bae JS. Sphingolipids in neuroinflammation: A potential target for diagnosis and therapy. BMB Rep. 2020;53(1):28–34. 31818364 10.5483/BMBRep.2020.53.1.278 PMC6999823

[62] Kosicek M , Hecimovic S. Phospholipids and Alzheimer's disease: Alterations, mechanisms and potential biomarkers. Int J Mol Sci. 2013;14(1):1310–1322. 23306153 10.3390/ijms14011310 PMC3565322

[63] Giussani P , Prinetti A, Tringali C. The role of Sphingolipids in myelination and myelin stability and their involvement in childhood and adult demyelinating disorders. J Neurochem. 2021;156(4):403–414. 33448358 10.1111/jnc.15133

[64] Wattenberg BW . Intra- and intercellular trafficking in sphingolipid metabolism in myelination. Adv Biol Regul. 2019;71:97–103. 30497846 10.1016/j.jbior.2018.11.002 PMC6469711

[65] Watanabe A , Kinouchi H, Horikoshi T, Uchida M, Ishigame K. Effect of intracranial pressure on the diameter of the optic nerve sheath. J Neurosurg. 2008;109(2):255–258. 18671637 10.3171/JNS/2008/109/8/0255

[66] Fonteh AN , Chiang J, Cipolla M, et al Alterations in cerebrospinal fluid glycerophospholipids and phospholipase A2 activity in Alzheimer's disease. J Lipid Res. 2013;54(10):2884–2897. 23868911 10.1194/jlr.M037622 PMC3770101

[67] Assi E , Cazzato D, De Palma C, Perrotta C, Clementi E, Cervia D. Sphingolipids and brain resident macrophages in neuroinflammation: An emerging aspect of nervous system pathology. Clin Dev Immunol. 2013;2013:309302. 24078816 10.1155/2013/309302 PMC3775448

[68] Liu Q , Zhang J. Lipid metabolism in Alzheimer's disease. Neurosci Bull. 2014;30(2):331–345. 24733655 10.1007/s12264-013-1410-3 PMC5562656

[69] Yin F , Sancheti H, Patil I, Cadenas E. Energy metabolism and inflammation in brain aging and Alzheimer's disease. Free Radic Biol Med. 2016;100:108–122. 27154981 10.1016/j.freeradbiomed.2016.04.200 PMC5094909

