# Supplementary material for: Determining the role of novel metabolic pathways in driving intracranial pressure reduction after weight loss
Source: Brain Commun. 2023 Oct 18;5(5):fcad272. doi: 10.1093/braincomms/fcad272 (PMC10608960; doi:10.1093/braincomms/fcad272)
Supplement: fcad272_Supplementary_Data [file fcad272_supplementary_data.zip › Supplementary Figures.pdf]

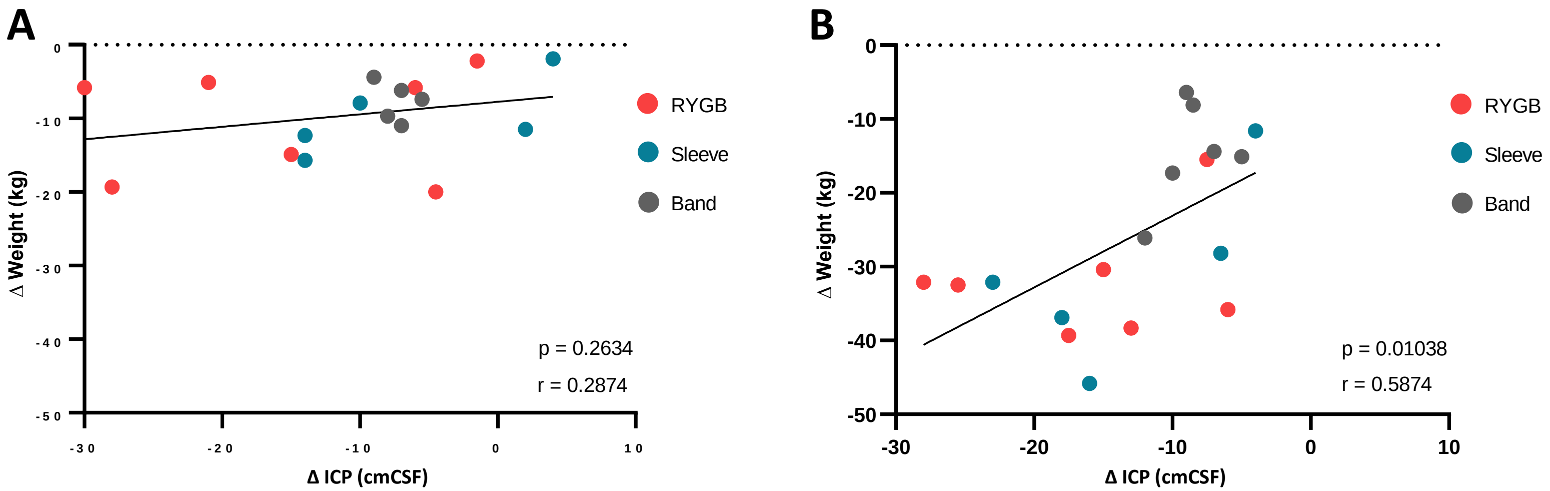

Supplementary Figure 1 Spearman rank correlation of Δ ICP (cmCSF) vs Δ Weight (kg) from baseline to 2 weeks post-surgery (A) and baseline to 12 months post-surgery (B) for each specific surgery group (RYGB (red) (*n* = 7), gastric sleeve (blue) (*n* = 5) and gastric banding (grey) (*n* = 6). (A) p = 0.2634, r = 0.2874; (B) p = 0.01038, r = 0.5874.

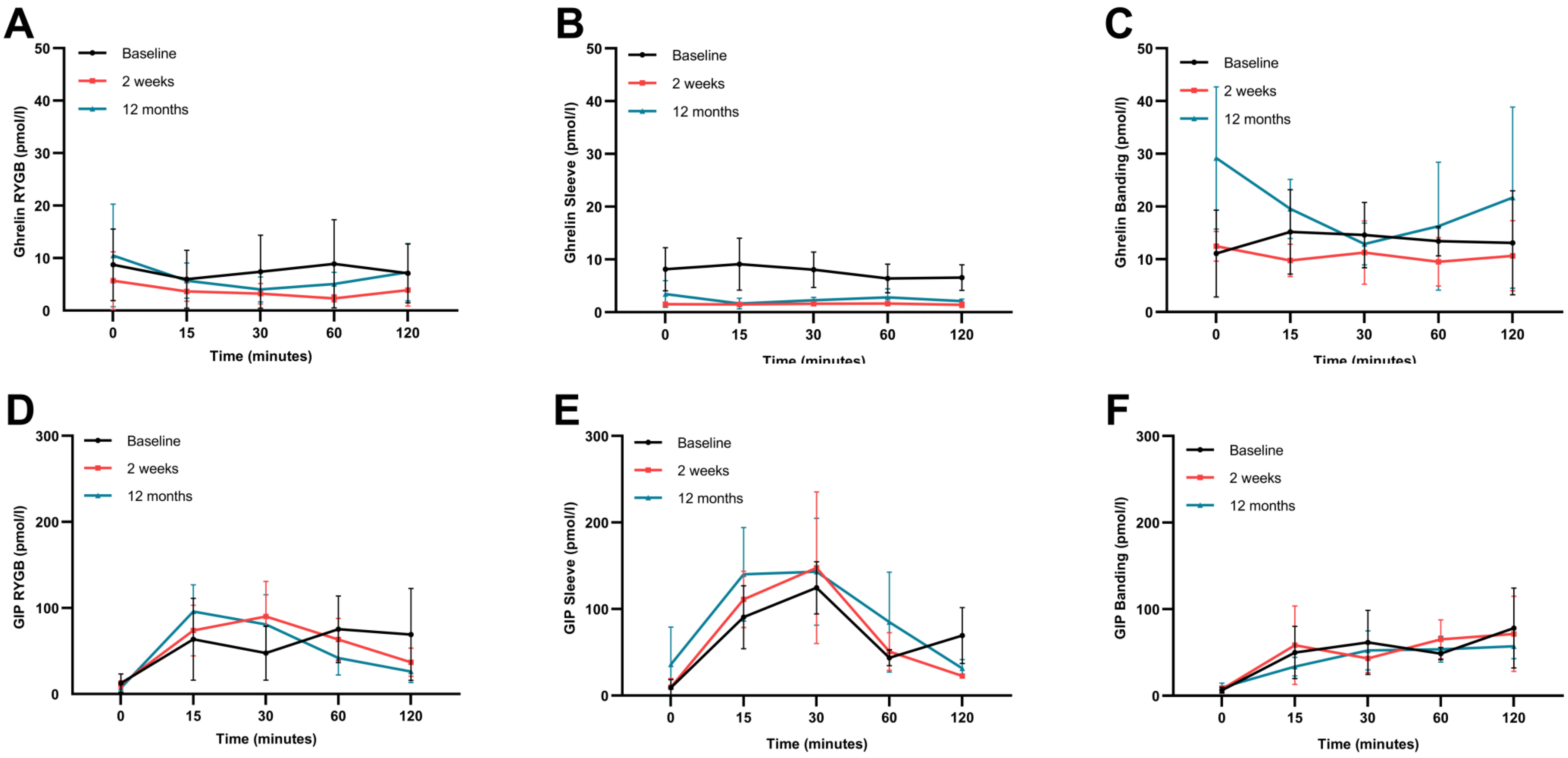

Supplementary Figure 2 Ghrelin and GIP gut hormone responses in all surgical cohorts at all time points. Total area under the curve dynamics of Ghrelin RYGB (Baseline Total Area: 934.6±356.9; 2 Weeks Total Area: 390.2±110.0, -58%; 12 Months Total Area: 702.5±201.2, -25%) (n = 7) (A); Ghrelin gastric sleeve (Baseline Total Area: 862.7±142.8; 2 Weeks Total Area: 186.1±20.63, -78%; 12 Months Total Area: 291.9±59.87, -66%) (n = 3) (B); Ghrelin gastric banding (Baseline Total Area: 1637±343.2; 2 Weeks Total Area: 1242±273.9, -24%; 12 Months Total Area: 2186±670.0, +34%) (n = 5) (C); GIP RYGB (Baseline Total Area: 7585±2186; 2 Weeks Total Area: 7171±1219, -5%; 12 Months Total Area: 5980±1016, -21%) (*n* = 7) (D); GIP gastric sleeve (Baseline Total Area: 8278±1198; 2 Weeks Total Area: 8032±1678, -3%; 12 Months Total Area: 10346±2315, +24%) (*n* = 3) (E); GIP gastric banding (Baseline Total Area: 6720±1567; 2 Weeks Total Area: 6983±1603, +4%; 12 Months Total Area: 5889±766.4, -12%) (*n* = 5) (F) following a meal stimulation at 0 to 120 minutes as total area ± standard error, and percentage change over baseline. Only descriptive analysis was performed on this figure. No formal statistical testing was performed. Total Area units = (pmol/l x minutes). Baseline: black lines; 2 weeks post-surgery: red lines; 12 months post-surgery: blue lines.
