# Supplementary material for: Determining the role of novel metabolic pathways in driving intracranial pressure reduction after weight loss
Source: Brain Commun. 2023 Oct 18;5(5):fcad272. doi: 10.1093/braincomms/fcad272 (PMC10608960; doi:10.1093/braincomms/fcad272)
Supplement: fcad272_Supplementary_Data [file fcad272_supplementary_data.zip › Supplementary Methods.pdf]

Determining the role of novel metabolic pathways in driving intracranial pressure reduction after weight loss.

Metabolomics analysis

Chemicals

Formic acid (≥98.0% purity), methanol (LC-MS grade), acetonitrile (HPLC grade) and isopropanol (HPLC grade) were purchased from VWR International (Lutterworth, U.K.) Ammonium formate (≥98.0% purity) and water (LC-MS grade) was purchased from Sigma-Aldrich (Poole, U.K.).

Metabolite extraction

Sample preparation was carried out as described previously.[1] Sample preparation order for all biological samples were randomised applying the RAND() function in Microsoft Excel. All samples were extracted during a single day by one scientist. All samples were thawed on wet ice, vortexed (15 seconds) and aliquoted (50µL) into clean 2mL Eppendorf tubes. For preparation of samples for HILIC analysis, 150 µL ice-cold 50/50 acetonitrile/methanol was added to the 50µL biological sample, the mixture was vortexed (15 s), centrifuged (20,000g, 20 min, 4°C) and 100 µL of supernatant was aliquoted into low recovery HPLC vials. For preparation of the lipids samples, the same procedure was followed as for HILIC, except that 150 µL of ice-cold 100 % isopropanol was used instead of 50/50 acetonitrile/methanol. A pooled QC sample was prepared by aliquoting 50µL of each biological sample in to a 15mL Falcon tube followed by vortex mixing

for 5 minutes. Multiple 50µL aliquots of the pooled QC sample were prepared as described above for HILIC and lipids analysis, each solution was analysed once. Two process (extraction) blank samples were prepared in exactly the same way as biological samples but in the absence of biological material.

Ultra High Performance Liquid Chromatography-Mass Spectrometry assays

The samples were maintained at 4°C and analysed applying two Ultra Performance Liquid Chromatography-Mass Spectrometry (UPLC-MS) methods using a Vanquish Liquid Chromatography System UPLC+ (Thermo Fisher Scientific, MA, USA) coupled with a heated electrospray Q Exactive Plus mass spectrometer (Thermo Fisher Scientific, MA, USA). Twelve pooled QC samples were analysed at the start of an analytical batch to condition the analytical system. Pooled QC samples were then analysed after every 6th biological sample and twice at the end of the analytical batch. The process (extraction) blank samples were analysed as injection 6 and as the last injection of the batch. All six biological samples collected over 120 minutes during the meal stimulation were analysed sequentially but the collection time was randomised using the RAND() function in Microsoft Excel. The analysis order in relation to patient and phenotype were randomised applying the RAND() function in Microsoft Excel.

Polar extracts were analysed on a Accucore-150-Amide-HILIC column (100 x 2.1 mm, 2.6 µm, Thermo Fisher Scientific, MA, USA). For positive ion mode analysis, mobile phase A consisted of 10 mM ammonium formate and 0.1% formic acid in 95% acetonitrile/water and mobile phase B consisted of 10 mM ammonium formate and 0.1% formic acid in 50% acetonitrile/water. For

negative ion mode analysis, mobile phase A consisted of 10 mM ammonium acetate and 0.1% acetic acid in 95% acetonitrile/water and mobile phase B consisted of 10 mM ammonium formate and 0.1% acetic acid in 50% acetonitrile/water. For both positive and negative ion mode the flow rate was set for 0.50 mL.min^{-1} with the following gradient: t=0.0, 1% B; t=2.1, 1% B; t=4.1, 15% B; t=7.1, 50% B; t=10.1, 95% B; t=11.0, 95% B; t=11.5, 1% B; t=15.0, 1% B, all changes were linear with curve = 5. The column temperature was set to 35°C and the injection volume was 2 µL. Data were acquired in positive and negative ionisation modes separately within the mass range of 70 – 1050 *m/z* at resolution 70,000 (FWHM at *m/z* 200). Ion source parameters were set as follows: Sheath gas = 55 arbitrary units, Aux gas = 14 arbitrary units, Sweep gas = 4 arbitrary units, Spray Voltage = 3.2kV (positive ion) / 2.7kV (negative ion), Capillary temp. = 380 °C, Aux gas heater temp. = 440°C. Data dependent MS2 in 'Discovery mode' was used for the MS/MS spectra acquisition applying a pooled QC sample for each sample type using the following settings: resolution = 17,500 (FWHM at *m/z* 200); Isolation width = 3.0 *m/z*; stepped collision energies (stepped CE) = 20, 40, 100 [positive ion mode] / 40, 60, 130 [negative ion mode]. Spectra were acquired in five different mass ranges with each range acquired for a separate QC sample injection (QC samples 6-10): 70 – 210 *m/z*; 200 – 310 *m/z*; 300 – 410 *m/z*; 400 – 510 *m/z*; 500 – 1050 *m/z*. A Thermo ExactiveTune 2.8 SP1 build 2806 was used as instrument control software in both cases and data were acquired in profile mode.

Non-polar (lipid) extracts were analysed on a Hypersil GOLD column (100 x 2.1mm, 1.9 µm; Thermo Fisher Scientific, MA, USA). Mobile phase A for positive and negative ion modes consisted of 10 mM ammonium formate and 0.1% formic acid in 60% acetonitrile/water and mobile phase B for positive and negative ion modes consisted of 10 mM ammonium formate and

0.1% formic acid in 90% propan-2-ol/water. Flow rate was set for 0.40 mL.min^{-1} with the following gradient: t=0.0, 20% B; t=1.6, 20% B, t=9.4, 100% B; t=10.6, 100% B; t=12.6, 20% B; t=15.0, 20% B, all changes were linear with curve = 5. The column temperature was set to 55 °C and the injection volume was 2µL. Data were acquired in positive and negative ionisation mode separately within the mass range of 150 – 2000 *m/z* at resolution 70,000 (FWHM at *m/z* 200). Ion source parameters were set as follows: Sheath gas = 48 arbitrary units, Aux gas = 15 arbitrary units, Sweep gas = 0 arbitrary units, Spray Voltage = 3.2kV (positive ion) / 2.7kV (negative ion), Capillary temp. = 380°C, Aux gas heater temp. = 450°C. Data dependent MS2 in 'Discovery mode' was used for the MS/MS spectra acquisition using following settings: resolution = 17,500 (FWHM at *m/z* 200); Isolation width = 3.0 *m/z*; stepped collision energies (stepped CE) = 20, 40, 100 [positive ion mode] / 40, 60, 130 [negative ion mode]. Spectra were acquired in five different mass ranges with each range acquired for a separate QC sample injection (QC samples 6-10): 150 – 510 *m/z*; 500 – 710 *m/z*; 700 – 860 *m/z*; 850 – 1010 *m/z*; 1000 – 2000 *m/z*. A Thermo ExactiveTune 2.8 SP1 build 2806 was used as instrument control software in both cases and data were acquired in profile mode. A Thermo ExactiveTune 2.8 SP1 build 2806 was used as an instrument control software in both cases and data were acquired in profile mode.

Raw data processing

Vendor format raw data files (.RAW) were converted to the mzML file format using ProteoWizard software.[2] Deconvolution was performed by the XCMS R package (version 3.12 running in R Version 4.0.5). XCMS was operated applying min peak width (6s); max peak width (30s); ppm (14); mzdiff (0.002); bw (0.25); mzwid (0.01); minfrac (0.2).. A data matrix of peak areas for metabolite features (*m/z*-retention time pairs) vs. samples were constructed for each of four assays.

Data for the first ten QC samples were removed from the dataset prior to further processing and analysis. Each data matrix was filtered as follows: re-equilibration QC samples (QC samples 1-8) were removed; any feature whose median intensity in the biological samples was <20× its median intensity of the extraction blank samples was removed; any feature present in < 70% of the QC samples was removed; features with RSD ≥ 30% across the pooled QC samples (QC9 to last QC sample analysed) were removed.

Metabolite annotation

Putative metabolite annotation applying MS1 data (defined as MS1 in Supplementary Tables) was performed by applying the Python package BEAMSpy (https://github.com/computational-metabolomics/beamspy). The parameters applied were maximum retention time = 2; grouping method = Spearman Rank (Coefficient threshold = 0.5, P-value threshold = 0.05); reference adduct, isotope and neutral loss databases were used; metabolite annotation based on *m/z* applied the Human Metabolome Database, Kegg - human and LIPID-MAPS with a mass tolerance of +/- 5ppm. Confirmation of metabolite annotations was performed applying matching of retention time to an in-house retention time library (RT error +/- 10s) (defined as MS1 AND RT in Supplementary Tables) and/or matching of MS/MS data to mzCloud (https://www.mzcloud.org; match >70%) (defined as MS/MS or MS/MS AND RT in Supplementary Tables). Also to generate robust lipid annotations, features within the QC sample UHPLC-MS/MS data were searched against an in-silico HCD MS/MS database using LipidSearch software (version 4.2.18, Thermo Fisher Scientific) (defined as MS/MS in Supplementary Tables). The precursor and product ion *m/z* tolerance was set to 5 ppm and potential ion forms used in the search included $[M+H]^+$, $[M+NH_4]^+$, $[M+Na]^+$, $[M+K]^+$, $[M+H-H_2O]^+$, $[M+2H]^{2+}$, $[M-H]^-$, $[M+HCOO]^-$, $[M+Cl]^-$, $[M-2H]^{2-}$

. Only annotations graded A-C were retained (Grade A – all fatty acyl chains and class were completely identified; Grade B – some fatty acyl chains and the class were identified; Grade C – either the lipid class or some fatty acyls were identified).

Statistical analysis

Gut neuropeptide: The gut neuropeptide data was analysed by calculating the total AUC from fasted samples at baseline, 2 weeks post-surgery and 12 month time points as well as from the time of ingestion of the mixed meal to the postprandial phase (0 to 120 min) at all time points. Statistical analysis was performed using GraphPad Prism 9.4.1 9 (GraphPad software).

Metabolomics: Statistical and pathway enrichment analysis applied to the metabolomics datasets was performed in MetaboAnalyst v5.0.[3] For statistical analysis, data were normalized to total sample response and log10 transformed. Statistical analysis applied two-way repeated measures ANOVA ($p<0.005$). Pathway enrichment analysis applied pathway analysis, hypergeometric test (enrichment method), relative-betweeness centrality (topology analysis) and *Mus musculus* (KEGG) as the pathway library. Fold change and +/-95% confidence intervals were calculated using the minimum or maximum peak response in comparison to the baseline sample (whichever was the higher) reported across the 120 minute period of collection post-meal. The mean was calculated for each class and the fold change was calculated by dividing the mean for class 1 by the mean for class 2. Any metabolites with a fold change between 0.9 and 1.1 were removed from the results files as less than 10% relative changes are viewed as inaccurate.

Metabolomics: For the correlation analysis of ICP with metabolites we performed Spearman Rank correlation analysis. The abundance of each metabolite detected (time point 0, the start of the meal stimulation test) were correlated with ICP measured at 2 weeks post-surgery. Separately, the changes in ICP (2 weeks post-surgery minus baseline values) were also compared to changes in abundances of each metabolite detected (fasted metabolites at time point 0 of the meal stimulation test). These comparisons were performed for RYGB only patients and for the combined set of sleeve and RYGB patients. Correlation of sleeve data was not performed because data was only available for three sleeve patients.

Metabolomics: Hierarchical Clustering Heatmaps were constructed in MetaboAnalyst using the following parameters; Features (autoscaled), distance measure (Euclidean), clustering method (Ward), samples (not clustered). The minimum or maximum peak response in comparison to the baseline sample (whichever was the higher) reported across the 120 minute period of collection post-meal were used.

Supplementary References

1. Southam AD, Haglington LD, Najdekr L, et al. Assessment of human plasma and urine sample preparation for reproducible and high-throughput UHPLC-MS clinical metabolic phenotyping. Analyst. 2020;145(20):6511-23.
2. Kessner D, Chambers M, Burke R, Agus D, Mallick P. ProteoWizard: open source software for rapid proteomics tools development. Bioinformatics. 2008;24(21):2534-6.
3. Pang Z, Chong J, Zhou G, et al. MetaboAnalyst 5.0: narrowing the gap between raw spectra and functional insights. Nucleic Acids Res. 2021;49(W1):W388-W96.
